# A reassessment of the early archaeological record at Leang Burung 2, a Late Pleistocene rock-shelter site on the Indonesian island of Sulawesi

**DOI:** 10.1371/journal.pone.0193025

**Published:** 2018-04-11

**Authors:** Adam Brumm, Budianto Hakim, Muhammad Ramli, Maxime Aubert, Gerrit D. van den Bergh, Bo Li, Basran Burhan, Andi Muhammad Saiful, Linda Siagian, Ratno Sardi, Andi Jusdi, Abdullah, Andi Pampang Mubarak, Mark W. Moore, Richard G. Roberts, Jian-xin Zhao, David McGahan, Brian G. Jones, Yinika Perston, Katherine Szabó, M. Irfan Mahmud, Kira Westaway, Jatmiko, E. Wahyu Saptomo, Sander van der Kaars, Rainer Grün, Rachel Wood, John Dodson, Michael J. Morwood

**Affiliations:** 1 Australian Research Centre for Human Evolution, Environmental Futures Research Institute, Griffith University, Brisbane, Queensland, Australia; 2 Balai Arkeologi Makassar, Makassar, Indonesia; 3 Balai Pelestarian Cagar Budaya, Jambi, Indonesia; 4 Place, Evolution and Rock Art Heritage Unit (PERAHU), Griffith University, Gold Coast, Queensland, Australia; 5 Centre for Archaeological Science, School of Earth & Environmental Sciences, University of Wollongong, Wollongong, New South Wales, Australia; 6 Independent Archaeologist, Makassar, Indonesia; 7 Museum Kepresidenan Republik Indonesia Balai Kirti, Paledang-Bogor, Indonesia; 8 Balai Pelestarian Cagar Budaya, Makassar, Indonesia; 9 Stone Tools and Cognition Hub, School of Humanities, Archaeology and Palaeoanthropology, University of New England, Armidale, New South Wales, Australia; 10 ARC Centre of Excellence for Australian Biodiversity and Heritage, University of Wollongong, Wollongong, New South Wales, Australia; 11 School of Earth and Environmental Sciences, University of Queensland, Brisbane, Queensland, Australia; 12 School of Earth & Environmental Sciences, University of Wollongong, Wollongong, New South Wales, Australia; 13 Department of Environmental Sciences, Macquarie University, Sydney, New South Wales, Australia; 14 Pusat Penelitian Arkeologi Nasional (ARKENAS), Jakarta, Indonesia; 15 Cluster Earth & Climate, Faculty of Earth and Life Sciences, Vrije Universiteit, Amsterdam, The Netherlands; 16 School of Earth, Atmosphere and Environment, Monash University, Clayton, Victoria, Australia; 17 Research School of Earth Sciences, The Australian National University, Canberra, Australian Capital Territory, Australia; 18 State Key Laboratory of Loess and Quaternary Geology, Institute of Earth Environment, Chinese Academy of Sciences, Xi'an, Yanta District, Shaanxi, China; Institucio Catalana de Recerca i Estudis Avancats, SPAIN

## Abstract

This paper presents a reassessment of the archaeological record at Leang Burung 2, a key early human occupation site in the Late Pleistocene of Southeast Asia. Excavated originally by Ian Glover in 1975, this limestone rock-shelter in the Maros karsts of Sulawesi, Indonesia, has long held significance in our understanding of early human dispersals into ‘Wallacea’, the vast zone of oceanic islands between continental Asia and Australia. We present new stratigraphic information and dating evidence from Leang Burung 2 collected during the course of our excavations at this site in 2007 and 2011–13. Our findings suggest that the classic Late Pleistocene modern human occupation sequence identified previously at Leang Burung 2, and proposed to span around 31,000 to 19,000 conventional ^14^C years BP (~35–24 ka cal BP), may actually represent an amalgam of reworked archaeological materials. Sources for cultural materials of mixed ages comprise breccias from the rear wall of the rock-shelter–remnants of older, eroded deposits dated to 35–23 ka cal BP–and cultural remains of early Holocene antiquity. Below the upper levels affected by the mass loss of Late Pleistocene deposits, our deep-trench excavations uncovered evidence for an earlier hominin presence at the site. These findings include fossils of now-extinct proboscideans and other ‘megafauna’ in stratified context, as well as a cobble-based stone artifact technology comparable to that produced by late Middle Pleistocene hominins elsewhere on Sulawesi.

## Introduction

**Fig 1:**
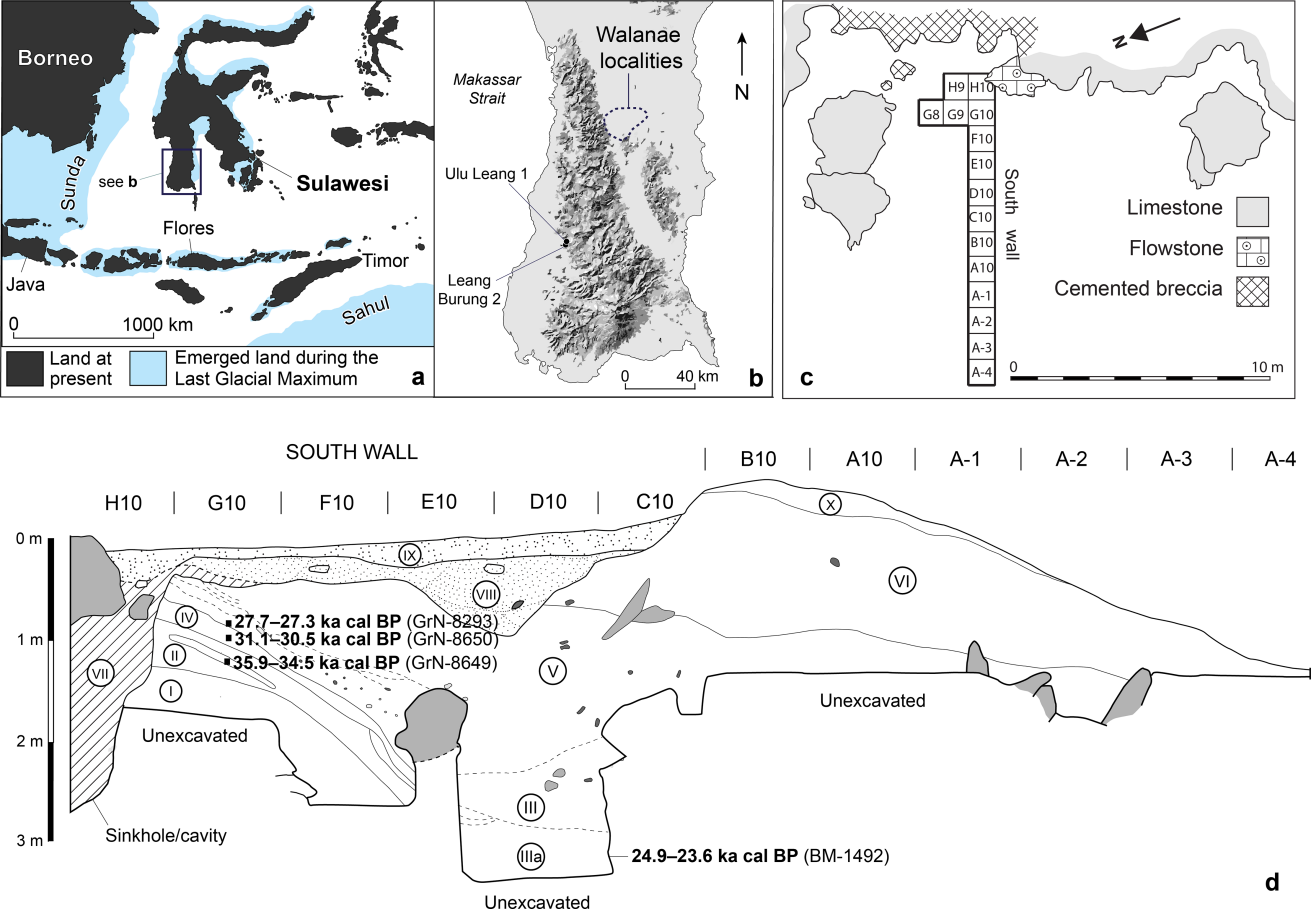
Study locality and context. Map of the Indonesian island of Sulawesi (**a**) showing the location of the Late Pleistocene rock-shelter site, Leang Burung 2 (**b**); the Holocene cave site, Ulu Leang 1, is located around 1.5 km to the north (**c**), plan view of Ian Glover’s 1975 excavations at Leang Burung 2; (**d**) stratigraphic profile of the south wall of the 1975 trench (redrawn from Fig 3 in [1]). Calibrated ^14^C ages are reported at the 95% confidence interval.

Leang Burung 2 is a limestone rock-shelter site [1] on the Indonesian island of Sulawesi, the largest and oldest island within the oceanic archipelago (‘Wallacea’) separating continental Asia (Sunda) from the Pleistocene low-sea level landmass of Australia-New Guinea (Sahul) (Fig 1). This Wallacean island, renowned for its unique biodiversity and particularly high rate of species endemism, for instance among mammals [2, 3], is generally assumed to have been a key stepping-stone on one of the two most likely early human dispersal routes from the edge of Sunda to the northern fringes of Sahul [4]. Modern humans (*Homo sapiens*) may have made first landfall on Sulawesi by 50,000 years ago (50 ka) [5], and perhaps by as early as 65 ka [6], based on early colonization dates for Australia.

Two recent archaeological discoveries have brought the early human prehistory of Sulawesi to world attention. First, uranium-series (U-series) dating of coralloid speleothems overlying hand stencils and animal paintings in the limestone karsts of Maros, in the south of the island [7], demonstrated that Sulawesi’s rock art dates back to at least 40 ka [8], and is thus compatible in age with the world’s earliest dated cave paintings, at El Castillo in northern Spain [9]. Second, deep-trench excavations at the open site of Talepu in the Walanae Depression, a fault-bounded sedimentary valley in the interior of the southern peninsula (Fig 1), revealed *in situ* stone artifacts in fossil-bearing strata dated to ~194–118 ka [10]).

The antiquity of the Talepu artifacts implies that the first colonizers of Sulawesi were either Asian *Homo erectus*, members of the *Homo floresiensis* lineage, Denisovans, or an as-yet unknown group of archaic hominins [10]. Concerning *H*. *floresiensis*, it has been hypothesized that the founding population that gave rise to this endemic Late Pleistocene hominin of Flores originated on Sulawesi, and thus that hominin occupation of the latter island dates back at least several hundred millennia [11]. It follows that the Talepu tool-makers may have been close evolutionary cousins of *H*. *floresiensis*. Alternatively, recent work suggests our species had emerged in northern Africa by ~300 ka [12], while some archaeological and genomic data imply that *H*. *sapiens* was established in eastern Asia [13], and possibly Sunda (Java; [14]), by 120 ka (see also [15, 16]. It is therefore at least conceivable, based on currently available evidence, that the early Middle Pleistocene inhabitants responsible for tool manufacture at Talepu were early members of our species that had dispersed from Africa and spread into Wallacea long before the Late Interglacial [10].

**Fig 2:**
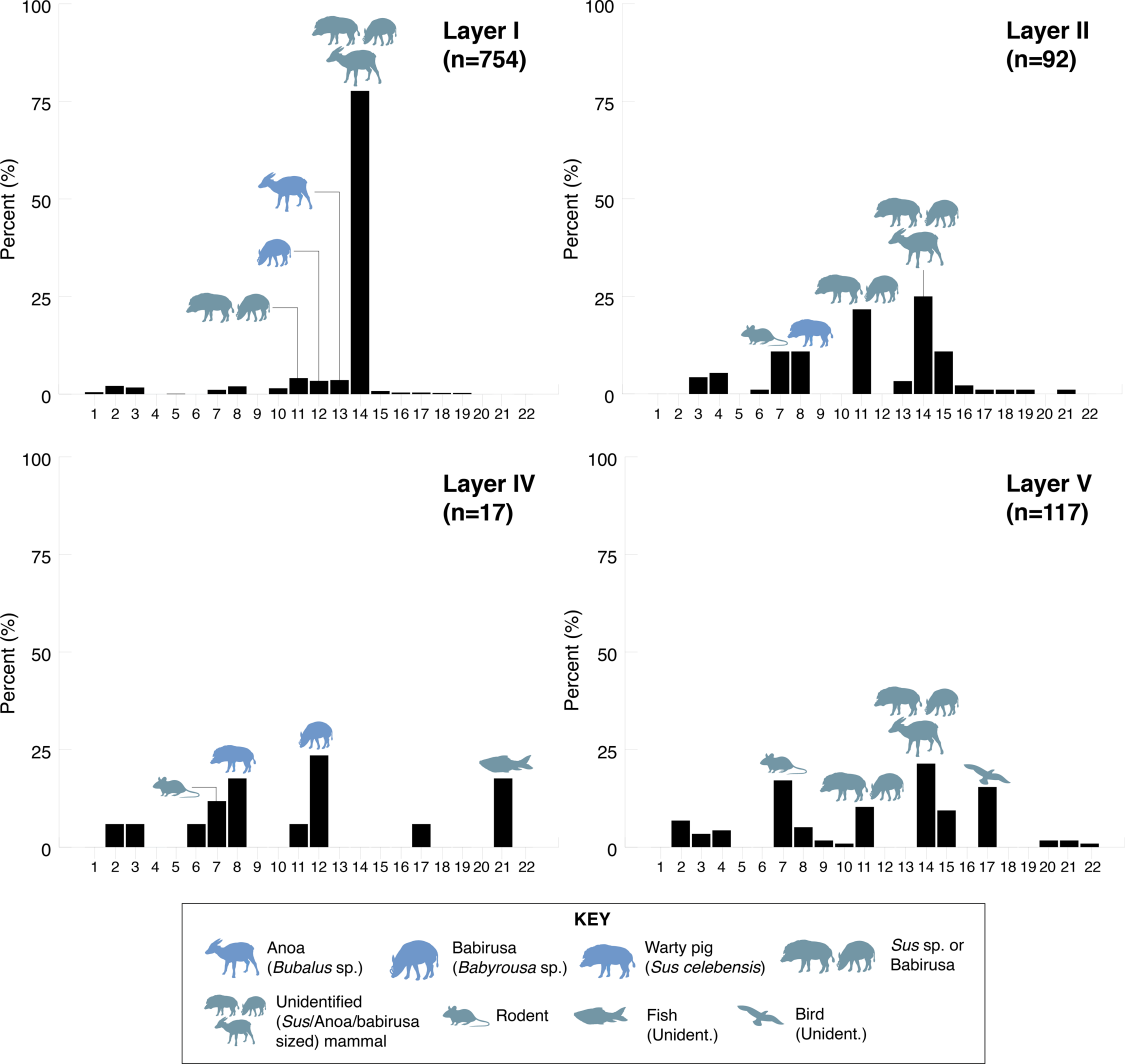
Faunal distributions recorded by Glover’s 1975 excavations at Leang Burung 2. Data source: Glover’s 1975 excavations [1], as reported by Clason [17]. Fauna codes: 1, *Strigocuscus celebensis* (ground cuscus); 2, *Ailurops ursinus* (bear cuscus); 3, flying fox; 4, *Macaca* sp. (macaque); 5, *Homo sapiens*; 6, Carnivora (unident.); 7, rodent; 8, *Sus celebensis* (Celebes warty pig); 9, *Sus*. sp; 10, *Sus*/Anoa; 11, *Sus*/babirusa; 12, Babirusa; 13, Anoa; 14, *Sus*/Babirusa/Anoa size (unidentified); 15, bear cuscus/macaque size (unidentified); 16, rodent size (unidentified); 17, bird (unidentified); 18, terrapin/tortoise; 19, reticulated python; 20, snake (unidentified); 21, fish (unidentified); 22, crayfish (unidentified). Clason’s [17] Table 1 lists Layer I and Layers IV-V as containing one and two *Sus scrofa* specimens, respectively. We follow Simons and Bulbeck [18] in regarding these identifications as implausible, and thus have included them in Clason’s [17] *Sus celebensis* tally for those layers. We also excluded Clason’s [17] "?" category, which dominates percentages for each layer. According to Table 1 in Clason’s [17] report, a single element attributed to *Homo sapiens* was identified in the Layer I faunal assemblage; however, this specimen is not described elsewhere in Clason’s [17] paper and there is no further reference in the literature to human skeletal material from Leang Burung 2.

Prior to these discoveries, the earliest record of a human presence on Sulawesi came from Ian Glover’s [1] excavations at the Maros rock-shelter Leang Burung 2 (Fig 1). Over the course of a single field season at this site in 1975, Glover excavated a series of 1 x 1 m squares, with the deepest reaching a depth of ~3.6 m. Excavations were discontinued before bedrock or sterile deposits were reached. Glover’s [1] earliest radiometric determination– 35.9–34.5 thousand calibrated radiocarbon years before present (35.9–34.5 ka cal BP [GrN-8649])–came from a sedimentary unit overlying a deep and then-undated layer of compact, yellow-brown clay at the trench base, Layer I (Fig 1). Immediately above Layer I, Glover [1] inferred the presence of a complex lithic technology (systematic macroblade manufacture) that, despite decades of subsequent research in Southeast Asia, remains unique in its temporal and regional context. Notably, excavations below this level in Layer I yielded cultural remains that contrasted sharply with those in overlying layers, inferred to span 31–19 thousand conventional ^14^C years BP, which when calibrated is around 35–24 ka cal BP [1]. The stone technology was different, and the Layer I fauna was dominated by two endemic and still-extant large-bodied mammals that were rare to absent in overlying deposits: the dwarf bovid anoa (*Bubalus* sp.), and the babirusa (*Babyrousa* sp.) [17, 18] (Fig 2).

It is also noteworthy that Glover’s [1] interpretation of the formation history of the site suggested that the Late Pleistocene deposit had been severely undermined by sink action, leading to significant slumping and deformation of archaeological layers across the excavated areas of the site. Glover’s work at Leang Burung 2, and wider research in Maros [19, 20], led him to conclude that extensive disturbances of caves and shelters had rendered the karsts a very challenging, and perhaps unproductive, region from an archaeological perspective. This may be at least part of the reason for the long hiatus in research efforts by non-Indonesian archaeologists in the limestone karsts of Maros from the mid-1970s until the early 2000s [21].

Glover [1] did not recover any Pleistocene human skeletal remains from Leang Burung 2 –nor did his excavations reach bedrock or culturally-sterile archaeological deposits. Concerning the latter, his excavations clearly raised the possibility that deeper excavations at Leang Burung 2 might reveal important new insight into the early human settlement of Sulawesi. For instance, plumbing the depths of Layer I, and any intact Pleistocene strata below, could reveal not only whether pre-modern hominins had indeed reached Sulawesi, but whether this population persisted on the island for long enough to have encountered early modern humans, and when and why these hominins became extinct or locally extirpated.

The cultural horizon in Layer I, assumed by Glover [1] to pre-date 40 ka, could also provide the first clues for understanding what happened to Sulawesi’s ‘megafauna’ once modern humans were introduced to this insular island ecosystem, with its unbalanced and highly distinctive suite of Wallacean land mammals [2, 3]. In the Walanae Depression, fossil-bearing strata of Early Pleistocene age have yielded remains of two distinct dwarfed or ‘pygmy’ proboscideans (*Stegodon sompoensis* and *Stegoloxodon celebensis*) and a large-sized *Stegodon* [22, 23]. By the Middle Pleistocene, however, only two proboscideans seem to have existed on Sulawesi, a large to intermediate-sized *Stegodon*, and an advanced, high-crowned elephant [23]. No well-documented traces of these taxa had ever been found in the Maros karsts or in secure contexts outside the fossil record of the Walanae Depression, so it had remained unclear when and how these proboscideans–and the island’s other extinct endemic ‘megafauna’ taxa, such as the archaic suid *Celebochoerus* [24]–had met their demise.

Despite the potential of Leang Burung 2, however, and notwithstanding the enduring importance of this site in regional archaeological syntheses (e.g. [25, 26]), most authorities have paid scant attention to the implications of the undated, culturally distinct occupation layer (Layer I) identified by Glover at the base of his 1975 trench (but see [18]).

In 2007, in an initial effort to redress this issue, and as part of a wider program of research into the evolutionary origins and dispersal of the *H*. *floresiensis* lineage, the late Professor Michael ‘Mike’ Morwood’s team conducted the first excavations at Leang Burung 2 in over three decades. These pilot investigations were followed by three separate and larger scale excavation seasons at Leang Burung 2, carried out between 2011 and 2013. The objectives of this new research program at the rock-shelter were as follows:

1. To reassess Glover’s [1] interpretation of the stratigraphic and cultural evidence in the uppermost deposits at Leang Burung 2, including his documentation of a distinctive Late Pleistocene occupation sequence spanning 35–24 ka cal BP, and to evaluate his model of site formation history that outlined evidence for large-scale undermining and warping of stratified deposits due to subterranean sinkhole action;
2. to vertically extend Glover’s 1975 excavations into Layer I in order to identify the base of this undated sedimentary unit and to establish a chronology for it;
3. in the event that Layer I did not terminate at bedrock, to continue excavations of deposits encountered below this layer down to bedrock or culturally-sterile strata in order to assess the record for stratigraphically earlier evidence of human habitation;

We report the results of our investigation in this paper.

### Research background

The limestone tower karst region of Maros and the adjoining Pangkep district to the north are located on an alluvial plain close to the western shoreline of Sulawesi’s southwestern peninsula [27] (Fig 1). Hereafter, we will refer to this karst landscape as a whole as Maros-Pangkep (or the Maros-Pangkep karsts), whereas individual karst valleys or regions (e.g., Maros) are named separately.

The karst lies between 4°7'S and 5°1'S and is formed within the Early/Middle Eocene to Middle Miocene Tonasa Formation [27]. The topography of this extensive (~400 km^2^) karst landscape is dominated by plateau-like hill masses formed by rivers cutting through intersecting joints in the limestone, and, in areas of advanced plateau dissection, classic steep-sided karst towers surrounded by alluvial plains that extend to the western coastline at an elevation of ~5 to 30 m a.s.l. [27]. The towers range from 1 to 10 km in diameter and 150 to 300 m in height. Extensive networks of footcaves developed around the bases as a result of subsurface weathering. These were exposed by lowering of the alluvial plains, or, alternatively, when uplift slowed or stopped long enough for laterally migrating rivers to undercut hillslopes [27, 28].

The archaeological potential of the multitude of caves and shelters in the vicinity of the Leang-Leang valley in the Maros karsts had been noted since the early 1900s [29]. Subsequent work prior to the outbreak of WWII, and in the final years of the Dutch colonial period, led to small-scale excavations at several Leang-Leang sites and at other localities in nearby karst regions [30–32] (see [33–35] for reviews). These early investigations brought to light the first traces of the so-named ‘Toalean’ culture of Maros and surrounding districts [31, 32]. The Toalean is a regionally unique industry of presumed middle to late Holocene antiquity that is characterized by backed blades, geometric microliths, and ‘Maros points’, small pressure-flaked projectiles with hollow bases and serrated margins [34, 36, 37].

The Dutch archaeologist, H.R. van Heekeren, visited Leang Burung 2 rock-shelter (called by him ‘Burung Cave’) in 1950, recording the presence of several hand stencils [31], and brecciated deposits on the rear wall that had been reported previously [38]. Thereafter, the karsts attracted little scientific interest until the Australian-Indonesian archaeological expedition to southern Sulawesi in 1969, led by J. Mulvaney and R.P. Soejono [35, 39]. These researchers excavated Leang Burung 1, a cave site located 150 m from Leang Burung 2, revealing a 4-m-deep sequence of Holocene deposits. Glover, also a member of this expedition, conducted a single excavation season at Leang Burung 2 as part of his own Maros-based field research program [1, 19, 20, 36], which concluded in the late 1970s.

**Fig 3:**
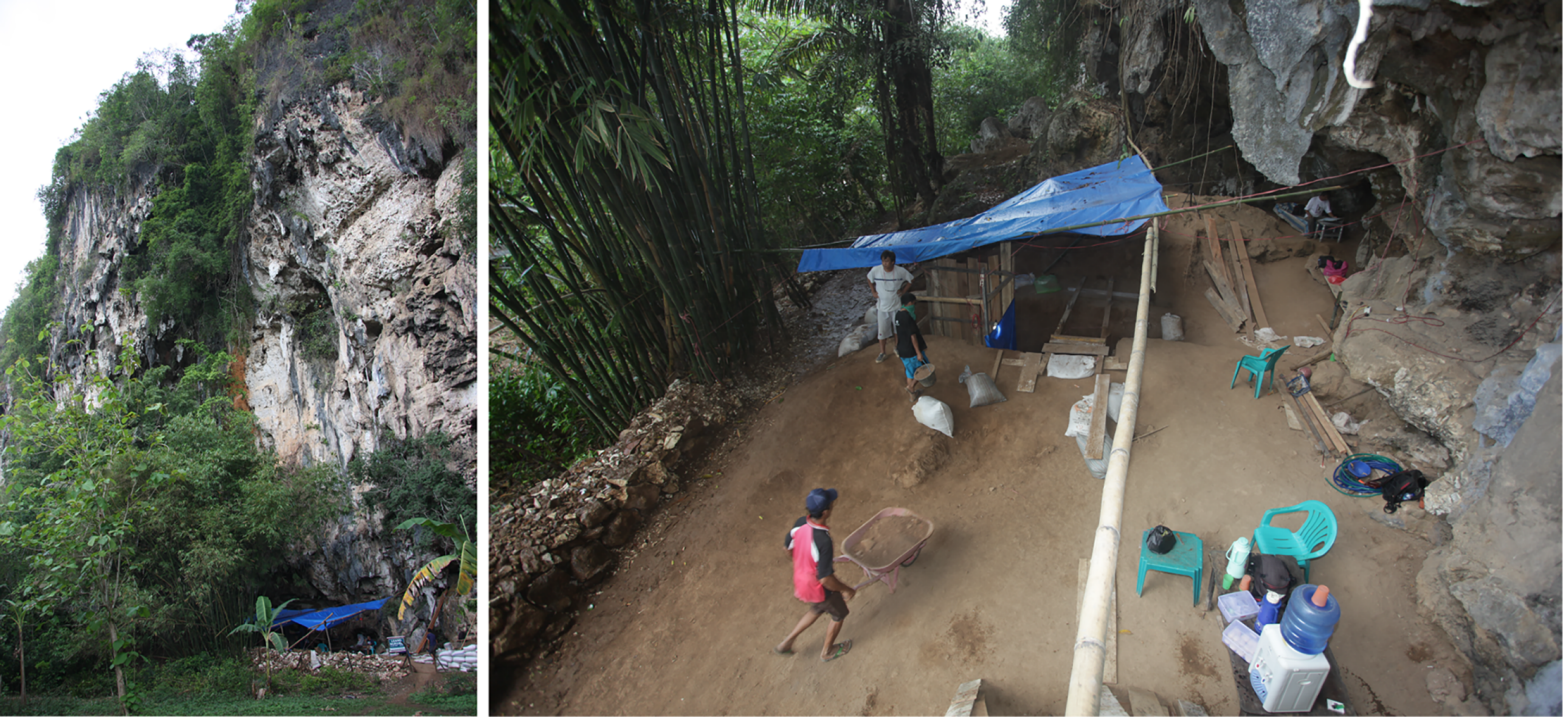
The cliff-foot rock-shelter at Leang Burung 2. Photographs taken during the 2011 excavation season.

Leang Burung 2 is one of many sheltered areas at the foot of tall, precipitous limestone cliffs lining the western flanks of a ~270 m-high karst tower, part of the main hill mass forming the southern side of the Leang-Leang river valley where it abuts the coastal plain of Maros (Fig 3). Located at the base of an 80 m high overhanging cliff face, the open, well-lighted site is 16 m long, 7 m wide, and about 20 m high, with a floor surface elevated 3 m above the level of the adjacent alluvial flats. The floor surface is marked by a large, shallow oval pit. According to Glover [1], this ‘robber’s hole’ was left by local people excavating phosphate-rich soil for sale as fertilizer, which took place in or around the year 1960.

**Fig 4:**
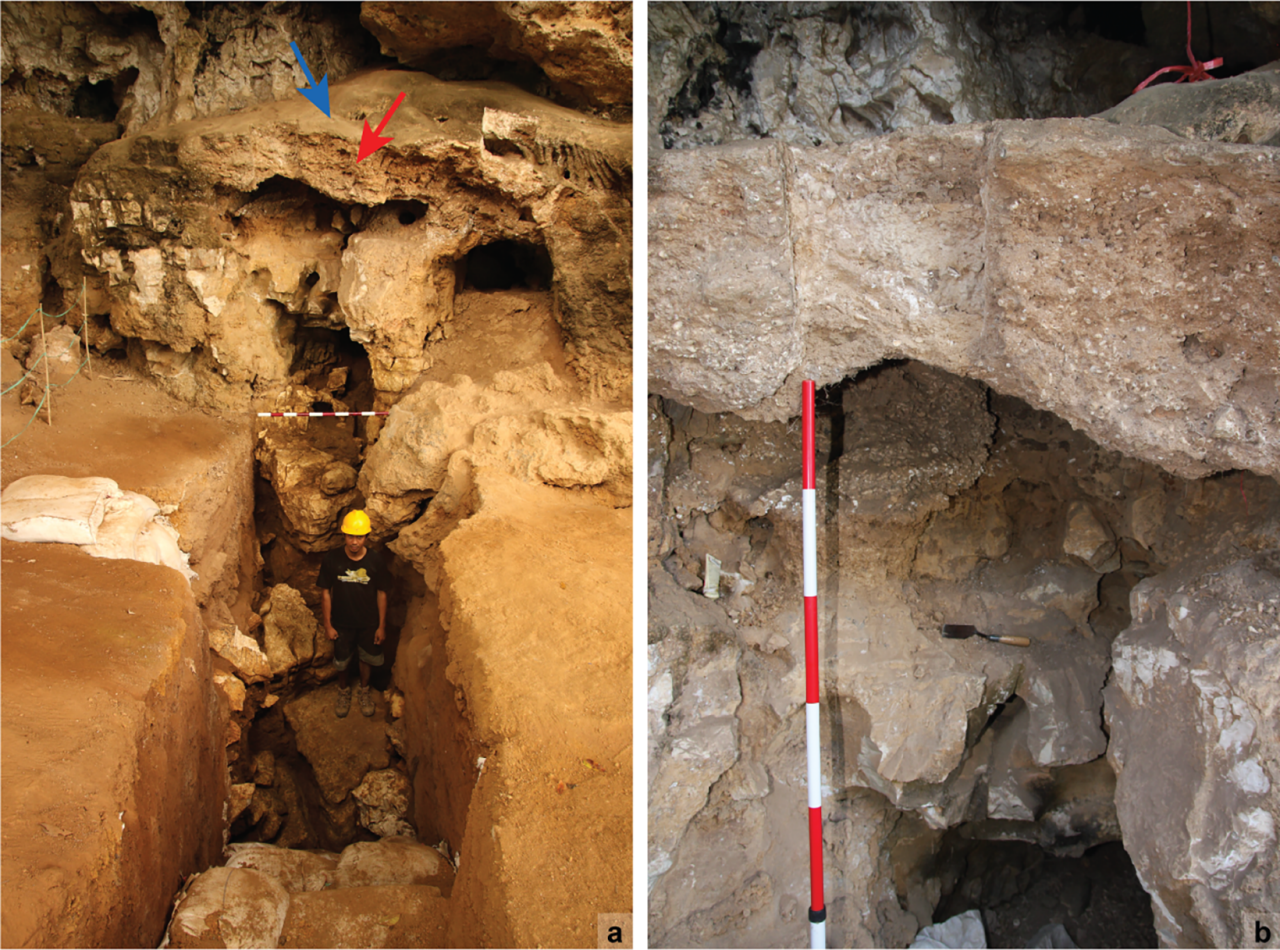
Cemented archaeological breccias on the rear wall of Leang Burung 2 shelter. (**a**) Viewed from towards the front of the shelter, looking from west to east along the 2013 trench–the red arrow points to the main brecciated mass; the blue arrow points to the capping flowstone; (**b**) close-up view of the highlighted area shown in panel **b**.

A notable feature of Leang Burung 2 –and many cave and shelter sites in the Maros-Pangkep karsts–is the presence of cemented archaeological deposits, or breccias, attached to the rear wall of the shelter, as well as to speleothem columns and some large roof-fall boulders exposed on the surface (often at the dripline; [1, 20]; see also [40]). On the rear wall of the shelter immediately adjacent to the excavated trench is a prominent bank of cemented breccias perched about 1.5 m above the present-day floor surface. This feature consists of a dense shell midden deposit capped by a thick layer of flowstone that slopes sharply downwards from the rear wall (Fig 4). Nearby, remnants of midden-bearing breccias adhere to the rear wall of the shelter at heights of up to 4 m above the ground surface.

### Glover’s 1975 excavations at Leang Burung 2

Glover’s [1] work at Leang Burung 2 in 1975 involved the excavation of 1 m^2^ units in a trench measuring 12 m in length by 1 m in width (Fig 1). The trench (~15 m^2^) extended southeast to northwest from near the rear wall to just past the dripline. The rearmost two squares adjacent to the wall were extended northeast, resulting in three additional 1 m^2^ units.

**Fig 5:**
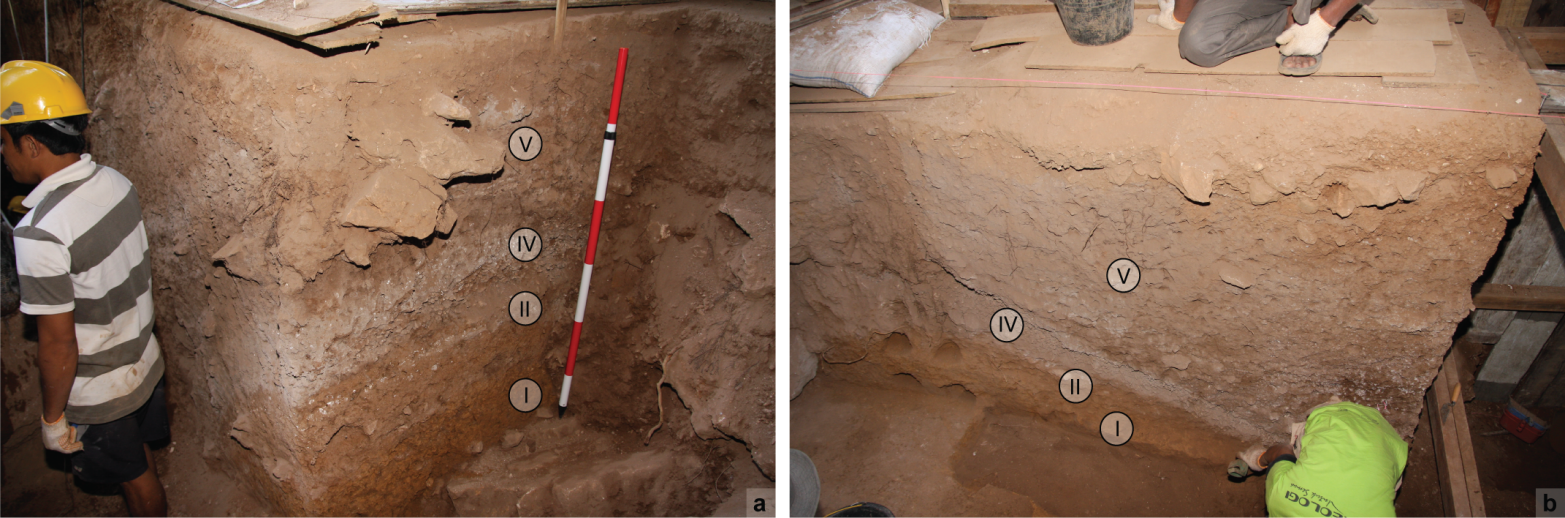
Stratigraphy revealed by Glover’s 1975 trench at Leang Burung 2. As viewed after emptying backfill in 2012; (**a**) north wall of square H9 and east wall of square G8; (**b**) south wall of Glover’s 1975 trench, squares E10 to G10.

From top to bottom, Glover’s [1] stratigraphic sequence (Fig 5) is as follows–*Layers X and IX and VIII*: recent shallow pit fills and thin deposits; *Layer VII*: rubbish-filled sinkhole deposit (~80 x 40 cm, depth >2.9 m); *Layer VI*: a sterile red earth up to 1.5 m thick [1]; *Layer V*: a ~200 cm thick series of brown/grey-brown/red-brown lenses and discontinuous inter-fingering layers; *Layer IV*: a thin (<20 cm) deposit of loose shells in an ashy matrix; *Layer II*: a 20–30 cm thick chocolate brown deposit containing few shells; *Layers III and IIIa*: massive, poorly defined deposits of reddish brown to yellow sediment (III) and chocolate brown sediment (IIIa), both of which contained few shells; and *Layer I*: compact yellow-brown clay with negligible shell content.

Glover [1] traced Layer V across nearly the whole trench. However, underlying Layers IV, II and I were only identified adjacent to the rear wall, whereas Layers III and IIIa, also inferred to underlie Layer V, were only documented in square D10. The deepest excavations were in squares F10 (2.5 m depth) and D10 (3.6 m depth). Glover was unable to reconcile the Layers IV/II/I stratigraphy exposed below Layer V in F10 with the sediments (Layers III/IIIa) also seen to underlie Layer V in D10. A large roof-fall block in E10 prevented him from joining up these two squares and thus clarifying a key part of the stratigraphy. In squares F10-H10 and G8-G10, Layer I was exposed to a depth of about 30 cm and over a surface area of ~4 m^2^. The lower boundary of this deposit was not revealed.

Charcoal from basal Layer VI (Fig 1) yielded a ^14^C age of 1708–1353 yr cal BP (I-9096) [1]. Glover obtained five ^14^C determinations on shells of the freshwater gastropod *Tylomelania* (= *Brotia*) *perfecta* from underlying strata [1]. *T*. *perfecta* shells from Layers V, IV and II in the south wall of square G10 returned in-sequence ages (at 95% probability) of 27.7–27.3 ka cal BP (GrN-8293), 31.1–30.5 ka cal BP (GrN-8650), and 35.9–34.5 ka cal BP (GrN-8649), respectively [1]. Two additional ^14^C ages on *T*. *perfecta* shells, one from the boundary between Layers IV and V (square H9), and another from Layer IIIa (square D10), yielded stratigraphically inconsistent ages of 32.6–31.4 ka cal BP (GrN-8292) and 24.9–23.6 ka cal BP (BM-1492), respectively [1]. No radiometric dates were obtained for Layer I owing to a lack of shell and other dateable materials.

Glover [1] claimed that ceramics and diagnostic Toalean tool types were absent below Layers IX, X, VIII and VII, and sterile Layer VI. Layers V, IV, II and III/IIIa yielded broadly similar lithic and faunal assemblages. Glover’s [1] analysis of the stone-flaking technology from these layers (see also [41]) indicates a relatively straightforward approach to lithic reduction based on hard-hammer knapping, and, less commonly, bipolar reduction. Small morphologically undifferentiated flakes dominated the assemblage. Very few flakes were retouched and only a small number of simple, intensively reduced cores (0.5%) were recovered. The most technologically and typologically distinctive implements in the assemblage were Levallois-like macroblades (n = 7) with facetted platforms, recovered *in situ* from Layers V, III and II [1]. Layer I yielded a total of 50 stone artifacts, only brief descriptions of which are published.

The most common stone type (96.7%) in Layers II, IV and V was high quality chert [1]. Lithic assemblages in Layers III and IIIa/b were also dominated by chert (95.7%). By contrast, 59% of artifacts in Layer I were manufactured from chert, with the proportions of limestone (31%) and quartz (10%) much higher than in overlying layers [1].

Glover [1] recovered ochre fragments from Layers II, IV-V, and V, including two utilized (faceted/scored) pieces in Layer V. Edge-glossed flakes (n = 31) were found in Layers II and above [1]. Wear traces on these artifacts were attributed processing of silica-rich plants for craft activities ([1]; see also [42]).

Glover’s [1] report provides limited detail on the faunal assemblages from Leang Burung 2. An accompanying paper [43] describes the shell analysis. Of the four edible species of shellfish in the deposit, *T*. *perfecta* [44] was dominant [43]. No evidence for exploitation of marine/estuarine species was reported [1, 43]. Layer I yielded negligible amounts of complete or even fragmentary shell [43, 45].

Vertebrate fossil remains at the site were generally in highly fragmentary condition, the majority were unidentifiable, and most exhibited signs of burning [17]. Most identifiable elements were in Layer I. Compared with Layer I, the assemblages in Layers II, IV, and V layers are notable for their high proportions of diagnostic *Sus celebensis* (Celebes warty pig) remains and rodent elements, and the minimal representation of identifiable elements of babirusas (n = 4, Layer IV) and anoas (n = 3, Layer II) (Fig 2).

Glover [1] postulated that Pleistocene deposits at Leang Burung 2 had undergone major post-depositional disturbances. He observed that the upper Layer I boundary and upper and lower boundaries of Layers II and IV dip sharply downwards (45°) from the rear of the shelter towards the front, ‘an inclination quite opposed to the trend of the modern surface and upper layers’ ([1], 11). In other areas he noted ‘the erratic and often sudden dipping of layers… (e.g. unit IIa…)’ ([1], 11). Excavations in H10 adjacent to the shelter wall also revealed the presence of ‘a sink developing below the deposit’ ([1], 7) (Fig 1). In addition, the presence of partly cemented archaeological deposits fixed to the rear wall of the shelter was interpreted to to indicate that a large portion of the uppermost stratigraphy had been scoured out.

Similar high-level cemented breccias were noted at Ulu Leang 1 [36], where patches of cemented breccias are located 1–2 m above the present floor surface [20]. Glover [36] initially hypothesized that an earlier sedimentary deposit had been all but rinsed out from Ulu Leang 1, leaving only remnants in the form of the cemented wall breccias. A subsequent phase of deposition then led to the accumulation of the soft floor deposits. However, ^14^C dating of charcoal from a patch of breccia located 70 cm above the present-day floor surface suggested to Glover [20] that the cemented deposit, and the upper part of the unconsolidated floor deposit below, were essentially the same age. Thus, he rejected the notion that there had been two separate phases of deposition [20], instead formulating the idea that erosion and undermining of the deposit from below, owing to karstic sink action, had led to subsidence. A similar process must have occurred at Leang Burung 2, Glover [1] argued. However, he inferred that the impact of karstic undermining on the Late Pleistocene strata at Leang Burung 2 had been more pronounced: ‘But, whereas at Ulu Leang 1 the deposits appear to have fallen evenly and parallel with the original surface, at Leang Burung 2 small sinks have formed in the cave floor, causing dramatic warping and local subsidence’ ([38], 311).

Glover [1] was unable to obtain any radiometric dates for the extensive high-level cemented breccias at Leang Burung 2. He claimed that samples of artifacts and faunal remains collected from these breccias were broadly similar to those excavated from the stratified floor deposits. No Toalean implements or ceramics were found. Noting that breccias were located at heights of up to 4 m above the shelter floor, Glover argued that they must represent the remnants of deposits that accumulated during the final phase of site occupation, and which had been almost completely scoured out by natural erosional processes–‘My expectation is that [the age of the breccias] should fall between 12000 and 8000 BP’ ([1], 17).

## Results

**Fig 6:**
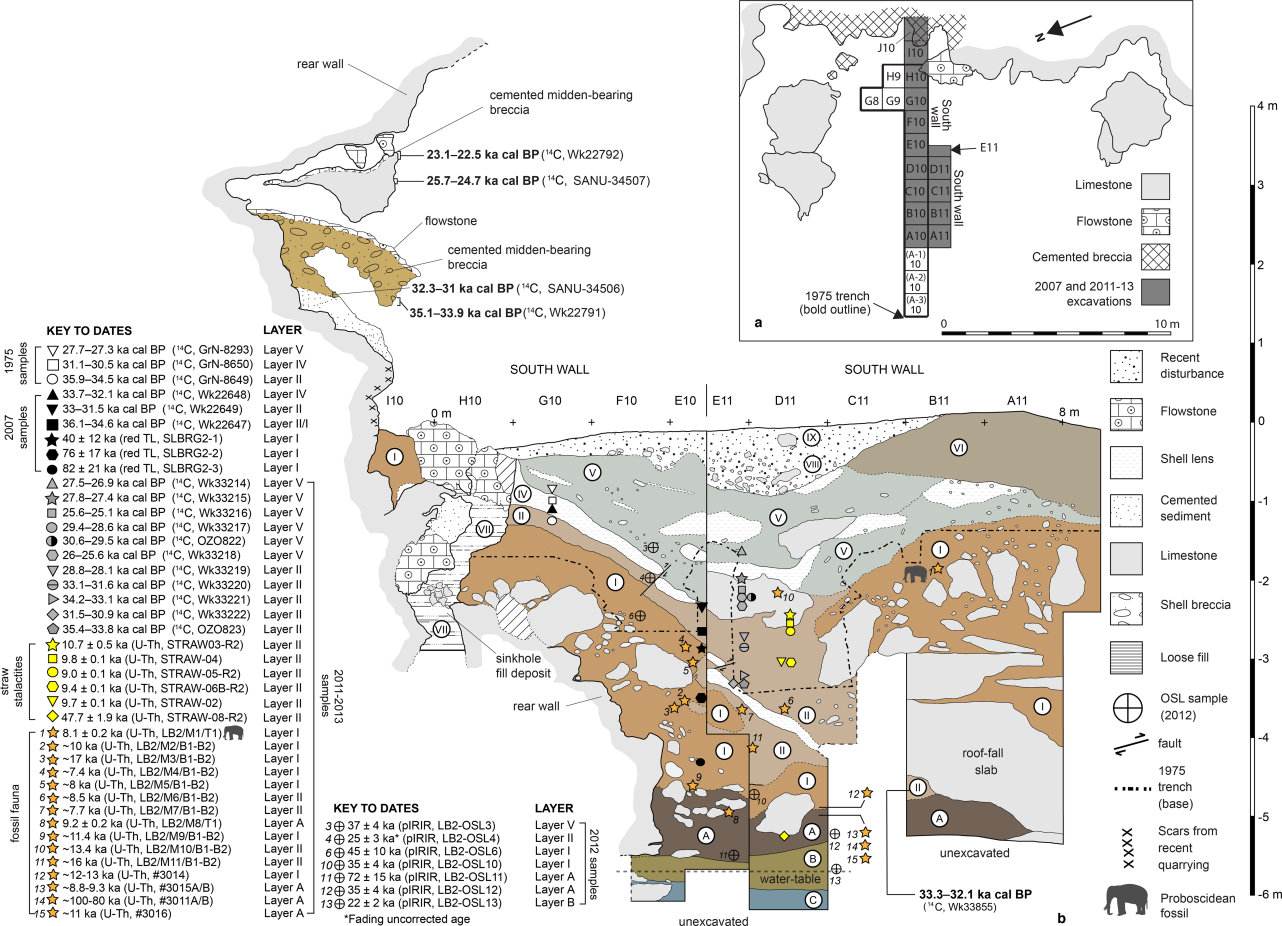
South wall stratigraphic profile at Leang Burung 2 (2011–13 excavations). The South Wall section illustrated here comprises a composite of two separate stratigraphic exposures, the southern trench faces of excavation squares E10-I10 and A11-E11, respectively, which are situated a distance of 1 m apart. With regards to ^14^C ages, all of the dated samples comprise freshwater gastropod (*Tylomelania perfecta*) shells; only dated shells collected *in situ* from the deposit are shown projected onto the stratigraphic profiles. The sampling locations for pIRIR samples LB2-OSL12 (Layer A) and LB2-OSL13 (Layer B) are not illustrated on the south wall profiles because they were collected from different trench faces.

Our pilot excavations at Leang Burung 2 in 2007 involved emptying backfill from squares E10 to G10 of the 1975 trench, where Glover [1] had obtained the oldest ^14^C date from the site and where he had traced Layer I to its deepest point ~2.6 m below the surface in the adjacent square F10. In 2011–13, our excavations below this level revealed the base of Layer I for the first time. Further deep-trench excavation over the course of these field seasons succeeded in extending Glover’s deepest excavated square (D10) from 3.6 m to 6.2 m depth (Fig 6). Excavations in 2011–13 also provided the opportunity to recover lithic and faunal assemblages from the uppermost strata and to evaluate Glover’s interpretation of the time depth of this distinctive occupation sequence. We will describe the results of the latter research first, then move to a discussion of our investigation of Layer I and underlying strata.

Archaeological and paleontological assemblages excavated from Leang Burung 2 in 2007 and between 2011 and 2013, and which form the basis of the current study, are under the permanent curation of Indonesian authorities at Pusat Penelitian Arkeologi Nasional (ARKENAS) in Jakarta and Makassar Balai Arkeologi. The material is available for analysis. Requests to access material for study, including databases and catalogs of finds, should be directed to the directors of ARKENAS and Balai Arkeologi Makassar.

### Evaluating Glover’s model of human occupation at the site 35–23 ka cal BP

**Fig 7:**
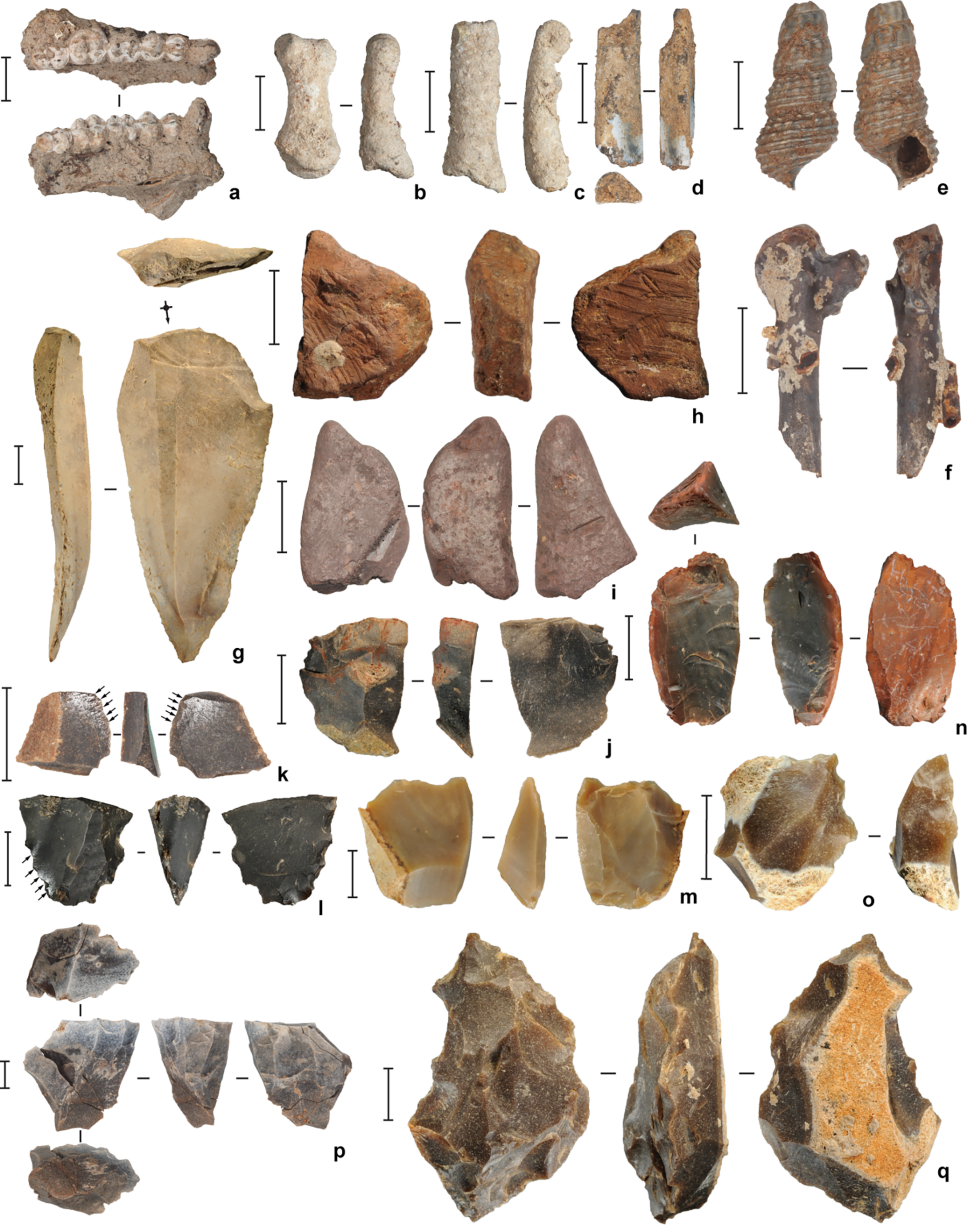
Faunal remains and artifacts excavated from the upper levels of square D11 at Leang Burung 2 in 2011.

Our 2011 excavations in squares A11-D11 (Fig 6) demonstrated that Glover’s [1] descriptions of the stratigraphic sequence and the archaeological inventory in the upper part of the deposit, below the recent disturbed layers, are broadly correct. We recovered the same types of findings reported by Glover [1] during our own excavations of Layers V, IV, II and III/IIIa-b (Fig 7). This includes: a chert-based lithic technology featuring edge-glossed flakes (Fig 7K and 7L) and macroblades (Fig 7G); evidence for pigment use (i.e., facetted and scored haematite pieces, and ochre residues on stone tools; Fig 7H and 7I); abundant signs of burning, as reflected by heat-affected stone artifacts, bones and shells, indicating an intensive use of fire; profuse quantities of freshwater gastropods (mostly *T*. *perfecta*), occurring in loose, midden-like concentrations (e.g., Layer IV) or as isolated findings; a relatively small, heavily fragmented vertebrate faunal assemblage characterized by a wide range of species, including *S*. *celebensis*, rodents, birds, bats, snakes, and freshwater aquatic fauna (i.e., fish, eels, crabs, and turtles)–of note was the predominance of bone fragments from small-bodied vertebrates, corresponding in size to monkeys (*Macaca* sp.; Fig 7A and 7D), cuscuses (*Ailurops* sp. and the smaller *Strigocuscus celebensis*) and civets (probably *Macrogalidia muschenbroeckii*). Based on our findings, we conclude that remains of larger-bodied mammals are far less common, and elements of anoas and babirusas, are very rare.

(**a**) *Macaca* sp. mandible, spit 31 (Layer II); (**b**-**c**) *Ailurops ursinus* (bear cuscus) phalanges; **b**, spit 42 (Layer II), **c**, spit 17 (Layer V); (**d**) *Macaca* sp. radius fragment (burnt), spit 46 (Layer II); (**e**) *Tylomelania* (= *Brotia*) *perfecta* shell (burnt), spit 19 (Layer II); (**f**) bird bone (burnt), spit 45 (Layer II); (**g**) chert macroblade point, spit 45 (Layer II); (**h**) utilised (faceted and scored) ochre piece, spit 45 (Layer II); (**i**) ochre ‘crayon’, spit 39 (Layer II); (**j**) chert flake with ochre residues, spit 30 (Layer II); (**k**-**l**) chert artifacts with silica gloss (highlighted by arrows); **k**, spit 18 (Layer V), **l**, spit 19 (Layer V); (**m**-**n**) chert bipolar cores; **m**, spit 19 (Layer V), **n**, spit 15 (Layer V); (**o**) retouched chert flake, spit 19 (Layer V); (**p**) single platform chert core, spit 45 (Layer II); (**q**) chert radial core, spit 13 (Layer V). Scale bars are 10 mm. Glover’s [1] excavations in square D10 had terminated in reddish brown Layer IIIa, which contained chert artifacts and shells, and thus was attributable to the same cultural sequence documented in overlying layers (assumed by Glover [1] to precede deposition of Layer I). Our excavations in squares D10-D11 showed that similar findings occurred in this deposit to a depth of nearly 5 m, at which point Layer I was encountered.

In 2012, by removing a series of large roof-fall blocks and a fallen stalactite that had obstructed Glover’s [1] excavations, we were able to show that the strata documented in 1975 at the base of square D10 (Layers III/IIIa-b) comprise a single deposit that is laterally continuous with Layer II to the east (Fig 6). We refer to this single, extensive sedimentary unit as Layer II. Layer II dips steeply downward from the rear wall of the shelter and starts to level out and thicken very considerably in the central floor area, before sloping up sharply and reducing in thickness to the west, where it eventually pinches out below Layer V (Fig 6). The thin, steeply dipping layer of loose shells contained in a grey ashy matrix (Layer IV) accumulated atop Layer II on the eastern slope of the channel-like trough. Layer V infills this depression and forms the topmost stratum within the sequence. Layer V is massive, but with numerous discrete and discontinuous shell lenses, rubble deposits, and other diffuse layers. Clasts (rock and shell) are imbricated and the sediments generally are poorly sorted, which may be suggestive of material slumping or washing downslope with varying degrees of flow intensity (slopewash deposition). Layer IV and II appear to have been compressed and plastically deformed in places by the impact of large roof-fall blocks collapsing from overhead cliffs onto the shelter floor, resulting in small faulted movements (Fig 6).

Based on the new stratigraphic details revealed by our 2011–13 excavations, it is now evident that Layer II overlies Layer I in the east (from the shelter wall to the central floor area) and Layer V in the west (at the front of the shelter) (Fig 6). There is a sharply defined boundary between Layer I and overlying Layer II, possibly suggesting a depositional hiatus separates these two units, while the interface between Layers V and I in the west is much less distinct. We also observed that Layer I occurs directly below Layer V in the westernmost part of the trench (squares A10-A11). It is now possible to interpret Layer II as a slopewash deposit that accumulated in a deep, channel-like depression or trough that had formed in underlying Layer I between the bedrock shelf and overlying roof-fall blocks near the rear wall and the massive block pile ‘dam’ fronting the shelter. In squares E10/11, we documented evidence for inter-fingering at the interface between Layers II and I. This observation suggests that during deposition of Layer II on the steeply sloping upper surface of underlying Layer I there was some sort of disturbance, such as a block fall or slump on slope, which resulted in wedge-like sections or lenses of Layer I becoming reworked and incorporated into Layer II. Such features may explain why Glover [1] reported the presence of a small number of finds characteristic of Layer II (i.e., chert implements) in the upper part of Layer I.

**Fig 8:**
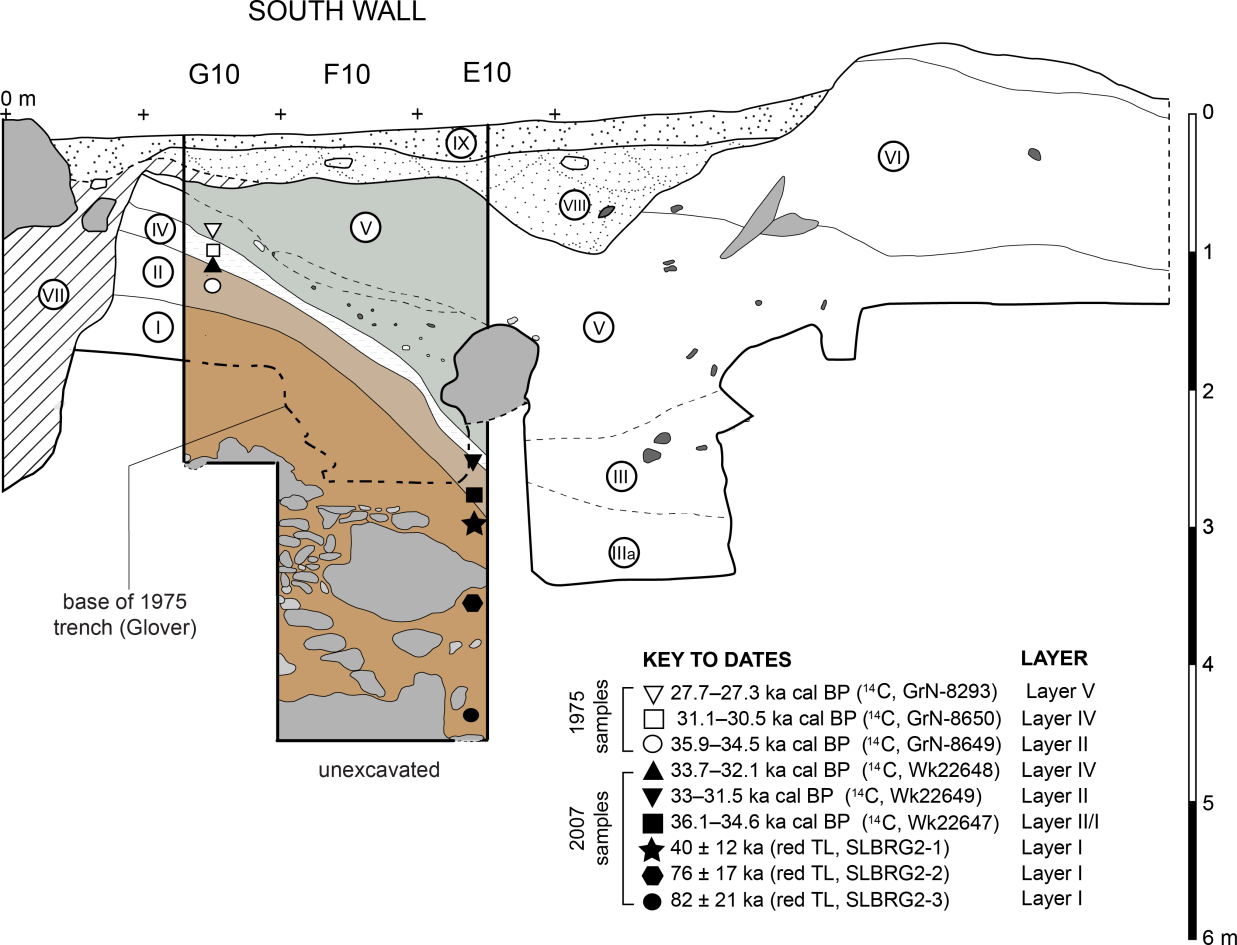
South wall stratigraphic profile at Leang Burung 2 (2007 excavations). Calibrated ^14^C ages are reported at the 95% confidence interval (see Table 1).The ^14^C dating results are presented in Table 1. All of the dated shells are Late Pleistocene in age, with two exceptions: a shell from Layer II (spit 48) yielded an unexpectedly young age of 9–8.6 ka cal BP (OZO824), while the suspected intrusive shell from Layer B (spit 58) was similar in age (9.6–9.5 ka cal BP, Wk33233). We regard both shells as having been displaced from stratigraphically higher contexts, most likely during the excavations.

We carried out an intensive program of radiometric dating in an effort to test the chronological framework proposed by Glover [1] for the upper levels (Layers V, IV and II). No charcoal or other carbonized plant material was recovered from the Leang Burung 2 deposits during the 2007/2011-13 excavations. Freshwater gastropod shells were the only materials available for radiocarbon dating (see Methods). As a result of the 2007 work, three accelerator mass spectrometry (AMS) ^14^C dates were obtained on fragments of freshwater shells collected from Layers IV and II, and at the interface between Layers II and I (Fig 8). Furthermore, a total of 23 new AMS ^14^C dates were run on freshwater shells (*T*. *perfecta*) recovered during the 2011 fieldwork season. Shells dated by the Radiocarbon Dating Laboratory at the University of Waikato (laboratory code prefix: Wk) and the AMS facility at the Australian Nuclear Science and Technology Organisation (ANSTO) (laboratory code prefix: OZ) were tested for secondary recrystallization using the Feigl method [46], while shells dated at the AMS facility at The Australian National University (laboratory code prefix: SANU) were analyzed using X-ray Diffraction (XRD) to check that the samples contained less than 0.3% calcite. Pretreatment, CO_2_ extraction and graphitisation methods for shells dated at ANSTO and The Australian National University followed procedures outlined in [47] and [48], respectively. Conventional ^14^C ages were calibrated using the ShCAL13 southern hemisphere calibration curve [49]. For further details on ^14^C dating methods see Table 1.

**Table 1:** Radiocarbon ages of shells and other materials from Leang Burung 2.

Year	Lab Code^a^^,^^b^^,^^c^	Layer	Sq	Spit/Depth below datum (BD)	Material	Conventional ^14^C age BP^d^	Calibrated age BP (95% confidence)^e^
1975	BM-1492	IIIa	D10	-	*Tylomelania perfecta*	20,150 ± 250	24,946–23,590
1975	GrN-8293	V	G10	-	*T*. *perfecta*	23,300 ± 140	27,744–27,275
1975	GrN-8650	IV	G10	-	*T*. *perfecta*	26,650 ± 200	31,131–30,505
1975	GrN-8292	IV-V	H9-1	-	*T*. *perfecta*	28,150 ± 200	32,629–31,400
1975	GrN-8649	II	G10	-	*T*. *perfecta*	31,260 ± 330	35,878–34,526
1975	I-9096	VI	A-2(10)	-	Charcoal	1,665 ± 80	1708–1353
2007	Wk22648	IV	G10	155 cm BD	Shell	28,907 ± 281	33,715–32,096
2007	Wk22649	II	E10	264 cm BD	Shell	28,386 ± 250	33,012–31,510
2007	Wk22647	II/I	E10	276 cm BD	Shell	31,415 ± 365	36,075–34,621
2011	Wk33214	V	D11	Spit 17	*T*. *perfecta*	22,917 ± 120	27,505–26,928
2011	Wk33215	V/II	D11	Spit 21	*T*. *perfecta*	23,406 ± 127	27,781–27,365
2011	Wk33216	V	D11	Spit 22	*T*. *perfecta*	21,051 ± 103	25,630–25,082
2011	Wk33217	V	D11	Spit 23	*T*. *perfecta*	24,928 ± 156	29,350–28,579
2011	OZO822	V	D11	Spit 23	*T*. *perfecta*	25,860 ± 200	30,623–29,488
2011	Wk33218	V	D11	Spit 24	*T*. *perfecta*	21,560 ± 103	26,014–25,616
2011	Wk33219	II	D11	Spit 28	*T*. *perfecta*	24,461 ± 148	28,786–28,076
2011	Wk33220	II	D11	Spit 29	*T*. *perfecta*	28,483 ± 238	33,136–31,602
2011	Wk33221	II	D11	Spit 33	*T*. *perfecta*	29,536 ± 276	34,162–33,060
2011	Wk33222	II	D11	Spit 34	*T*. *perfecta*	27,323 ± 206	31,478–30,931
2011	OZO823	II	D11	Spit 34	*T*. *perfecta*	30,620 ± 430	35,414–33,845
2011	Wk33223	II	D11	Spit 37^g^	*T*. *perfecta*	29,636 ± 273	34,262–33,198
2011	Wk33227	II	D11	Spit 40^g^	*T*. *perfecta*	30,581 ± 310	35,065–33,955
2011	Wk33228	II	D11	Spit 42^g^	*T*. *perfecta*	25,346 ± 163	29,813–28,909
2011	Wk33229	II	D11	Spit 44^g^	*T*. *perfecta*	25,254 ± 166	29,680–28,829
2011	Wk33224	II	D11	Spit 45^g^	*T*. *perfecta*	31,015 ± 329	35,613–34,260
2011	Wk33225	II	D11	Spit 46^g^	*T*. *perfecta*	28,570 ± 240	33,285–31,695
2011	Wk33226	II	D11	Spit 47^g^	*T*. *perfecta*	27,984 ± 241	32,535–31,243
2011	OZO824	II	D11	Spit 48	*T*. *perfecta*	7990 ± 70	8998–8605
2011	Wk33230	II	D11	Spit 49^g^	*T*. *perfecta*	29,555 ± 271	34,174–33,095
2011	Wk33855	II	B11	~540 cm BD	*T*. *perfecta*	28,693 ± 140	33,294–32,110
2007	Wk22792	Br^f^	-	3.4 m above floor level	Shell	18,987 ± 80	23,056–22,537
2012	SANU-34507	Br^f^	-	3.2 m above floor level	*T*. *perfecta*	20,990 ± 160	25,685–24,747
2012	SANU-34506	Br^f^	-	1.6 m above floor level	*T*. *perfecta*	27,640 ± 320	32,345–30,985
2007	Wk22791	Br^f^	-	1.5 m above floor level	Shell	30,597 ± 323	35,114–33,946

a Shells dated by the Radiocarbon Dating Laboratory at the University of Waikato (laboratory code prefix: Wk) were processed as follows (F. Petchey, pers. comm.): physical pretreatment involved cleaning surfaces and washing the samples in an ultrasonic bath; the samples were tested for secondary recrystallization using the Feigl method [46]. With regards to the latter, staining showed that all the shells were aragonite, with the exception of Wk22648 and Wk22649; both of these samples collected in 2007 contained a mixture of calcitic and aragonitic shells, with the change to calcite in some instances possibly owing to burning–the calcitic shells were removed and only those identified as aragonite by the staining test were selected for ^14^C dating. Chemical pretreatment involved acid washing with 0.1N HCl, after which the samples were rinsed and dried.

b Shells dated by the AMS facility at the Australian Nuclear Science and Technology Organisation (ANSTO) (laboratory code prefix: OZ) were pretreated as follows (F. Bertuch, pers. comm.): samples were cut in half and surface-drilled to remove any dirt/secondary carbonate material, after which they were ultrasonicated in water for two periods of 5 min duration in order to remove dirt/dust; this was followed by a surface etch by ultrasonicating the shells in 0.5M HCl for 2 min (50–80% of surface removed) and staining with Feigl solution [46]–the shells turned entirely black, indicating that they were composed of aragonite and no secondary calcite was present–staining was removed from shells with dilute HCl and then rinsing with water; shells were then loaded into hydrolysis vials with H_3_PO_4_, the vial evacuated, and then a further 10 min acid etch was performed, with the resulting CO_2_ discarded (to ensure no extraneous carbon was picked up during processing); the sample was then completely hydrolysed; finally, CO_2_ was extracted and then graphitized by reducing the CO_2_ over an iron catalyst (600°C) using hydrogen. Pretreatment, CO_2_ extraction and graphitisation methods followed procedures outlined in [47].

c Shells dated by the AMS facility at The Australian National University (laboratory code prefix: SANU) were pre-treated as follows: to remove potentially altered material, the surface and any soft material was removed with a drill, and 10% weight was removed in HCl. XRD was used to check that the samples contained less than 0.3% calcite and the clean material was graphitized and dated by AMS following procedures outlined in [48]. SANU-34507 contains <0.3% calcite. In the case of SANU-34506, however, XRD showed that the cleaned carbonate contained 0.7 ± 0.3% calcite. This suggests that the resultant ^14^C age determination might be slightly affected by the calcite (i.e., the date may be too young)–we note, however, that the date for SANU-34506 is stratigraphically consistent with bracketing ^14^C ages.

d Uncertainties reported at 95% confidence interval.

e Conventional ^14^C ages were calibrated using the ShCAL13 southern hemisphere calibration curve [49].

f Br: Brecciated deposit affixed to rear wall of shelter.

g Dated shell collected from wet-sieve residue.

Of the 23 new AMS ^14^C dates run on freshwater shells (*T*. *perfecta*), 21 shells derived from the excavated floor deposits, while two were collected from the brecciated wall deposits. Shells sampled from the excavations came from square D11, with the exception of one specimen, which was collected from below a roof-fall slab in square B11. We dated a total of six shells from Layer V and 15 shells from underlying Layer II. The assemblage of 21 dated shells spans a vertical distance of about 3.4 m, with the highest dated specimen in the sequence occurring at 1.6–1.7 m below the surface in Layer V, and the lowest dated specimen at ~5 m depth near the bottom of Layer II. We also dated a shell from Layer B which we suspected at this point was intrusive. For the first time, we also obtained radiometric dates for the cemented bank of breccia suspended on the wall above the shelter floor, carrying out AMS ^14^C dating on four freshwater shells collected from these remnant archaeological deposits.

Combined together, the three sets of radiocarbon dates on freshwater shells now available for the stratified deposits excavated at Leang Burung 2 in 1975, 2007 and 2011–13 display a range of age reversals and irregularities (Table 1). The shells from the stratigraphically youngest layer (V) are generally younger than those from the stratigraphically oldest layer (II). However, a pattern of age inversions is evident within and between the various depositional horizons. For instance, two shells collected from spits 23–24 in Layer V (~220–240 cm below surface) yielded inverted ^14^C ages of 30.6–29.5 ka cal BP (OZO822) and 26–25.6 ka cal BP (Wk33218), respectively. Dates on two shells (Wk33228 and Wk33229) from Layer II (spits 42–44) fall within broadly the same temporal range (~29 ka cal BP) of dated shells (Wk33217 and OZO822) from spit 23 in overlying Layer V. The similarity in age is despite these two sets of specimens being separated from each other in the deposit by a vertical distance of ~200 cm.

In contrast, ^14^C determinations on shells from the breccias display a coherent pattern of increasing age with depth (Table 1). A freshwater shell collected (in 2007) from near the base of a breccia outcrop 368 cm east of square F10, and at a height of 150 cm above the present-day floor surface of the shelter, yielded an age of 35.1–33.9 ka cal BP (Wk22791), while a shell from a breccia located higher up on the face of the rock wall (340 cm above the floor and 160 cm south of square F10) was dated to 23.1–22.5 ka cal BP (Wk22792). These results indicate a Late Pleistocene antiquity for the cemented breccias, demonstrating that they are substantially older than Glover [1] had anticipated (see above). Indeed, the ^14^C chronology from the breccias provides the first evidence that Glover’s [1] hypothesis about the age of these cemented deposits on the rear wall is in need of reassessment: rather than being younger than the ^14^C-dated floor deposits far below, the breccias fall within broadly the same time range.

With regards to the ^14^C chronology, the pattern of age reversals in the shells from the excavated floor deposit may be explicable given the depositional context. *T*. *perfecta* shells are small, highly mobile objects that are susceptible to post-depositional movement from one stratigraphic context to another. Shell midden deposits are also complex features prone to processes of deflation, erosion, and natural and anthropogenic disturbances that may lead to the conflation of shells of mixed age populations into a single deposit (i.e., time-averaging) and the redistribution of midden materials in archaeological sites [50]. Hence, a plausible argument can be made that the pattern of age reversals is owing to, say, deflation of nearby middens of Late Pleistocene antiquity and the subsequent redeposition of shells of different ages into Layers V, IV and II, which were accumulating within the shelter over roughly the same time period. However, this interpretation is inconsistent with the observation that ^14^C determinations on shells from the high-level breccias on the rear wall of the shelter in immediate proximity to the excavated trench display a coherent pattern of increasing age with depth (Table 1). Attaining a larger sample of dated shells from the breccias could alter this picture; for now, however, dated shells from the breccia display a positive age to depth relationship that is not evident in the excavated archaeological deposits–an observation that requires an explanation.

Also noteworthy is the close correlation between the shell-based ^14^C chronology of the brecciated deposits on the rear wall and the temporal range of shells from Layers V, IV and II (Table 1). It remains uncertain precisely when the remnant brecciated deposits were lithified or cemented *in situ*; that is to say, it is clear that the dated shells were present in sediments abutting the rear wall between approximately 35 and 23 ka cal BP, but when in time these deposits became indurated by the flow or seepage of calcium carbonate-enriched water is unknown.

The pattern of age reversals in the dated shells (Table 1) and the presence of a cemented, shell-rich deposits falling within precisely the same time range on the rear wall immediately above the shelter floor, raises two possibilities: (1) the rear wall breccias were previously laterally continuous with Layers V, IV and II within the shelter floor, and the portion of the deposit that once connected these units was subsequently lost due to erosion or recent anthropogenic disturbances (e.g., soil extraction by locals). This scenario would imply that the oldest shell-bearing strata at Leang Burung 2 (Layers II and V) once comprised a steeply inclined accumulation of midden deposits akin to the sloping flank of a massive shell mound (e.g., [51]), which had banked up against the shelter wall and attained a vertical height of at least 7 m; (2) the breccias represent the remnants of an older series of floor deposits that accumulated ~35–23 ka cal BP and were afterwards scoured out of the site by erosion; thereafter, formation of the Layers II-V stratigraphy commenced in the shelter floor and involved the incorporation of redeposited shells and other materials eroding from the breccias.

According to the second scenario–the basic premise of which Glover [1] had, in fact, initially considered and rejected as a possibility (see also [40])–the earliest phase of deposition of sediments comprising the stratigraphically oldest layer within the dated sequence of floor deposits (Layer II) would post-date 23.1–22.5 ka cal BP (Wk22792), which is the latest known formation phase of the cemented breccias on the wall. In other words, archaeological deposits at Leang Burung 2 long thought to date to between 31,000 to 19,000 conventional ^14^C years BP (35–24 ka cal BP) on the basis of Glover’s [1] investigations, could be post-LGM in age.

In an attempt to clarify the depositional ages of the *in situ* rock-shelter deposits, we conducted the post-infrared infrared stimulated luminescence (pIRIR_290_) dating procedure on feldspar samples collected in 2012 from Layer II (LB2-OSL4) and Layer V (LB2-OSL3). Mineralogy analysis of the sediments from Leang Burung 2 suggests that a high percentage of sanidine feldspars in the mineral composition of the sediments from this site. Thus, for the internal dose rate calculation, a K concentration of 10 ± 2% was assumed based on the theoretical K concentration of 10.69% for sanidine [52, 53], while a Rb concentration of 400 ± 100 ppm was assumed [54]. For further information on the pIRIR_290_ dating procedure used, see the Methods section and Table 2.

**Table 2:** Burial depths, grain sizes, water contents, dosimetry data, D_e_ and pIRIR ages for the feldspar samples collected from Leang Burung 2.

Sample	Depth	Grain size (μm)	Water Content (%)^a^	Gamma dose rate (Gy/ka)	Ext. beta dose rate (Gy/ka)	Int. beta dose rate (Gy/ka)^b^	Cosmic ray (Gy/ka)	Total dose rate (Gy/ka)	D_e_ (Gy)^a^		Age (ka)	
									Fading uncorr.	Fading corr.	Fading uncorr.	Fading corr.
LB2-OSL3 (Layer V)	160	180–212	20 ± 5 (18)	0.782 ± 0.012	1.115 ± 0.076	0.67 ± 0.15	0.08 ± 0.01	2.65 ± 0.12	103.6 ± 7.9	105 ± 5	37 ± 4	37 ± 4
LB2-OSL4^c^ (Layer II)	197	180–212	20 ± 5 (20)	0.807 ± 0.009	1.025 ± 0.09	0.67 ± 0.15	0.08 ± 0.01	2.58 ± 0.13	70.8 ± 7.6	-	25 ± 3	-
LB2-OSL6 (Layer I)	248	90–180	20 ± 5 (20)	1.216 ± 0.007	1.290 ± 0.084	0.48 ± 0.13	0.07 ± 0.01	3.06 ± 0.12	47.9 ± 10.1	148 ± 38	13 ± 3	45 ± 10
LB2-OSL10 (Layer I)	478	180–212	20 ± 5 (20)	1.028 ± 0.013	1.156 ± 0.074	0.67 ± 0.15	0.06 ± 0.01	2.91 ± 0.12	61.0 ± 6.7	111 ± 22	18 ± 2	35 ± 4
LB2-OSL11 (Layer A)	536	90–212	20 ± 5 (12)	1.063 ± 0.010	0.915 ± 0.070	0.53 ± 0.15	0.05 ± 0.01	2.57 ± 0.14	43.7 ± 8.3	195 ± 48	14 ± 3	72 ± 15
LB2-OSL12 (Layer A)	532	90–212	50 ± 10 (54)	1.275 ± 0.005	2.070 ± 0.190	0.53 ± 0.15	0.05 ± 0.01	3.94 ± 0.23	110.0 ± 10.1	147 ± 21	26 ± 3	35 ± 4
LB2-OSL13 (Layer B)	563	180–212	50 ± 10 (48)	2.380 ± 0.010	2.270 ± 0.230	0.67 ± 0.15	0.05 ± 0.01	5.38 ± 0.25	112.1 ± 8.8	128 ± 11	19 ± 2	22 ± 2

a The water content of 20 ± 5% was estimated based on field values shown in the brackets.

b Mineralogy analysis of the sediments from Leang Burung 2 suggests that a high percentage of sanidine feldspars in the mineral composition of the sediments from this site. Thus, for the internal dose rate calculation, a K concentration of 10 ± 2% was assumed based on the theoretical K concentration of 10.69% for sanidine [52, 53], while a Rb concentration of 400 ± 100 ppm was assumed [54].

c The equivalent doses were estimated using the sensitivity-corrected pIRIR 290°C signal. All the equivalent doses were corrected for residual doses obtained from bleached aliquots (see text). A fading test was not conducted on LB2-OSL4, and hence the fading-corrected D_e_ values are not available for this sample.

The luminescence dating results are presented in Table 2. Similar to the ^14^C chronology, the sequence of pIRIR age assessments generated from the 2012 samples is stratigraphically inverted. The fading corrected age for the topmost sample (LB2-OSL3) is 37 ± 3 ka, whereas the underlying sample from Layer II (LB2-OSL4) yielded a fading uncorrected age of 25 ± 3 ka. The fading corrected age (37 ± 3 ka) for the topmost sample, LB2-OSL3, collected from Layer V, is inconsistent with the hypothesis that this layer caps a sequence of strata (Layers II-V) that accumulated after deposition of the stratigraphically youngest ^14^C-dated shell (23.1–22.5 ka cal BP, Wk22792) from the overhead breccias. The anomalous result for LB2-OSL3 may be owing to insufficient bleaching of the dated feldspar grains. We did not conduct a fading test on LB2-OSL4 (Layer II), so the fading-uncorrected age of ~25 ka should be regarded as a minimum age–one that is also inconsistent with the aforementioned hypothesis.

We also undertook laser ablation U-series dating of faunal remains (N = 4) from Layer II, and solution U-series dating of five straw stalactites (‘soda straws’) recovered *in situ* from this deposit (see Methods). Results are presented in Tables 3 and 4, respectively. The earliest minimum U-series age for a fossil from Layer II is ~16 ka, while the youngest minimum age is 7.7 ± 0.2 ka (Table 3). The straw stalactites from Layer II are consistently early Holocene in age, with estimates ranging from ~10.2 to 8.4 ka. U-series dates for straw stalactites should generally be regarded as maximum ages for the stratigraphic contexts in which the samples are found [55]. However, owing to their rapid growth and limited life cycles, straw stalactites may also potentially provide ages that are close to the time of sediment deposition [55].

**Table 3:** Laser ablation U-series dating results for fossil faunal remains from Leang Burung 2.

Sample#^a^	Layer	Depth (cm)	U (ppm)	^230^Th/^238^U	^234^U/^238^U	CS Age (ka)
LB2/M10/B1 LB2/M10/B2	II	210–220 cm^b^	90.2 ± 14.9 94.1 ± 29.0	0.1400 ± 0.0042 0.1370 ± 0.0035	1.1943 ± 0.0085 1.1947 ± 0.0083	13.6 ± 0.4 13.2 ± 0.4
LB2/M6/B1 LB2/M6/B1	II	365 cm^c^	171 ± 9 164 ± 9	0.0896 ± 0.0019 0.0909 ± 0.0019	1.1983 ± 0.0079 1.1994 ± 0.0083	8.5 ± 0.2 8.6 ± 0.2
LB2/M7/B1 LB2/M7/B2	II	369 cm^c^	122 ± 7 129 ± 6	0.0815 ± 0.0020 0.0812 ± 0.0021	1.1903 ± 0.0082 1.1904 ± 0.0080	7.7 ± 0.2 7.7 ± 0.2
LB2/M11/B1 LB2/M11/B2	II	410–420 cm^b^	125 ± 38 126 ± 39	0.1641 ± 0.0034 0.1646 ± 0.0035	1.1973 ± 0.0079 1.1934 ± 0.0081	16.0 ± 0.4 16.1 ± 0.4
LB2/M1/T1	I	180–190 cm^b^	206 ± 87	0.0865 ± 0.0018	1.2031 ± 0.0078	8.1 ± 0.2
LB2/M4/B1 LB2/M4/B2	I	280–290 cm^b^	319 ± 6 325 ± 6	0.0762 ± 0.0016 0.0778 ± 0.0016	1.1757 ± 0.0076 1.1764 ± 0.0075	7.3 ± 0.2 7.5 ± 0.2
LB2/M5/B1 LB2/M5/B2	I	307 cm^c^	210 ± 22 226 ± 20	0.0870 ± 0.0018 0.0829 ± 0.0018	1.1705 ± 0.0077 1.1736 ± 0.0080	8.4 ± 0.2 8.0 ± 0.2
LB2/M2/B1 LB2/M2/B2	I	356 cm^c^	141 ± 10 134 ± 16	0.1047 ± 0.0022 0.1073 ± 0.0023	1.1789 ± 0.0081 1.1778 ± 0.0077	10.1 ± 0.2 10.4 ± 0.2
LB2/M3/B1 LB2/M3/B2	I	365 cm^c^	114 ± 10 112 + 14	0.1693 ± 0.0035 0.1723 ± 0.0035	1.1701 ± 0.0076 1.1708 ± 0.0076	17.0 ± 0.4 17.3 ± 0.4
LB2/M9/B1 LB2/M9/B2	I	460–470 cm^b^	114 ± 2 111 ± 8	0.1157 ± 0.0024 0.1170 ± 0.0025	1.1700 ± 0.0077 1.1707 ± 0.0076	11.3 ± 0.3 11.5 + 0.3
3014 Dentine 3014 Enamel	I	489 cm^c^	362±16 10.3±14.1	0.1229 ± 0.0020 0.1111 ± 0.0050	1.0635 ± 0.0057 1.0545 ± 0.0137	13.4 ± 0.2 12.1 ± 0.6
3015A 3015B	A	494 cm^c^	296 ± 14 380 ± 35	0.0820 ± 0.0015 0.0858 ± 0.0015	1.0517 ± 0.0058 1.0512 ± 0.0057	8.8 ± 0.2 9.3 ± 0.2
LB2/M8/T1	A	495 cm^c^	143 ± 42	0.0965 ± 0.0020	1.1890 ± 0.0078	9.2 ± 0.2
3011A Dentine 3011A Enamel 3011B Dentine 3011B Enamel	A	490–500 cm^b^	111 ± 18 10.1 ± 7.8 112 ± 7 1.73 ± 0.77	0.6729 ± 0.0111 0.4000 ± 0.0106 0.6200 ± 0.0100 0.4897 ± 0.0433	1.1329 ± 0.0066 1.1470 ± 0.0084 1.1323 ± 0.0062 1.1014 ± 0.0183	96.0 ± 2.7 46.3 ± 1.6 84.8 ± 2.2 63.4 ± 7.7
3016A 3016B	A	500–510 cm^b^	339 ± 40 446 ± 23	0.1035 ± 0.0017 0.1013 ± 0.0027	1.0537 ± 0.0059 1.0533 ± 0.0060	11.3 ± 0.2 11 ± 0.3

a **Sampled materials:** #3011A-B (Suidae molar encased within thick calcrete nodule); #3014–16 (Suidae molar); LB2/M1/T1 (proboscidean lamella fragment); LB2/M2/B1-B2 (Suidae mandible fragment [joint] partly encased within thick calcium carbonate/sandy matrix); LB2/M3/B1-B2 (proximal femur or tibia diaphysis fragment from a large mammal—‘pygmy’ proboscidean?); LB2/M4/B1-B2 (long bone fragment from large to medium-sized mammal; partly encrusted with thick covering of hard beige matrix/carbonate); LB2/M5/B1-B2 (distal ulna fragment, probably pig; whitish colour, partly covered with hard beige matrix); LB2/M6/B1-B2 (proximal humerus diaphysis fragment from small-sized mammal, possibly *Ailurops ursinus*; brown-greyish, encrusted, poor preservation; burnt. Treated with acetic acid); LB2/M7/B1-B2 (radius of possible *Macaca* sp. or *A*. *ursinus*—small to medium-sized mammal); LB2/M8/T1 (*Bubalus* sp. molar [lope]; partly encrusted with hard sandy beige-coloured matrix); LB2/M9/B1-B2 (humerus fragment, Suidae); LB2/M10/B1-B20 (skull fragment of unknown taxa); LB2/M11/B1-B2 (unidentified long bone fragment from a medium-sized mammal).

b Sample either excavated *in situ* by 10 cm-thick spit, but not 3D-plotted, or recovered from wet-sieve residue from that spit.

c Sample location/depth 3D-plotted *in situ*.

**Table 4:** Results of U-series dating of straw stalactites from Leang Burung 2.

Sample Name	Context (layer)	U (ppm)	(^230^Th/ ^232^Th)	Uncorr. Age (ka)	± 2σ	Corr. Age (ka)	± 2σ	Corr. Initial (^234^U/ ^238^U)	± 2σ
STRAW-02	Layer II	4.7612	60.68	9.816	0.048	9.7	0.1	1.0532	0.0010
STRAW-03-R1	Layer II	3.3805	9.30	11.592	0.078	10.6	0.5	1.0539	0.0009
STRAW-03-R2	Layer II	3.3368	9.69	11.606	0.055	10.7	0.5	1.0524	0.0010
STRAW-04	Layer II	3.4314	50.80	9.924	0.070	9.8	0.1	1.0526	0.0009
STRAW-05-R1	Layer II	4.5906	33.82	8.740	0.054	8.5	0.1	1.0528	0.0010
STRAW-05-R2	Layer II	4.2797	36.89	9.208	0.051	9.0	0.1	1.0534	0.0008
STRAW-06B-R1	Layer II	2.5580	141.42	9.340	0.078	9.3	0.1	1.0478	0.0010
STRAW-06B-R2	Layer II	2.5776	37.61	9.564	0.091	9.4	0.1	1.0487	0.0012
STRAW-08-R1	Layer A	0.5119	10.10	50.8	0.3	47.5	1.7	1.0146	0.0014
STRAW-08-R2	Layer A	0.5050	9.14	51.4	0.3	47.7	1.9	1.0155	0.0013
STRAW-06A	Layer II	4.5049	150.81	9.770	0.058	9.7	0.1	1.0445	0.0006

U-series age assessments for the straw stalactites are consistent with the hypothesis that the deposition of Layer II and overlying strata post-dates the erosion and mass loss of an older series of unconsolidated archaeological deposits at the site that spanned around 35–23 ka cal BP, and which are now represented only by cemented breccias adhering to the the rear wall of the shelter. Furthermore, the straw stalactite chronology suggests that deposition of Layer II commenced sometime after about 10.2 ka. There is a discrepancy between the oldest minimum U-series ages for bone fragments from Layer II (LB2/M10/B1-2 and LB2/M11/B1-2–13.6 ± 0.4/13.2 ± 0.4 ka and ~16 ka, respectively) and the postulated timeframe of <10.2 ka for the initial accumulation of Layer II. This suggests that the erosion and mass loss of archaeological deposits spanning 35–23 ka cal BP took place between approximately 13.6–13.2 ka and 10.2 ka.

### Depth, age, and cultural contents of Layer I

Based on our excavations in 2007 and 2011–13, it now evident that Glover’s Layer I is a massive weathered soil profile that is at least 3 m thick and which accumulated against the rear wall of the rock-shelter and atop a >2 m-high pile of limestone roof-fall slabs and angular blocks at the front of the shelter (Fig 6). Layer I directly abuts the rear wall of the rock-shelter from a depth of around 5 m to, essentially, the modern ground surface of the shelter. This deposit can be traced laterally from the shelter wall to the dripline. Near to the rear wall, this sedimentary unit is yellowish brown at the top and becomes conspicuously browner and more clayey with depth, with the former representing the weathered upper zone of the latter. The presence of clay-rich pockets or lenses within the upper zone is probably due to deposition in wetter conditions, leading to greater compaction of the sediments. Layer I contains large amounts of small, highly fragmentary bones and teeth, but very few stone artifacts. A sandy yellow interval at the base of Layer I adjacent to the rear wall contains a dense concentration of fossil fauna, including anoa molars and a near-complete babirusa upper incisor.

Layer I yielded a small assemblage of minimally reduced limestone and volcanic flakes. Ochre and shells, furthermore, are absent, and the faunal assemblages, while dominated by bone fragments from vertebrate mammals equivalent in size to macaques and *A*. *ursinus*, features relatively high proportions of remains from larger-sized animals, including diagnostic elements from babirusas and anoas. Remains from the latter mammals, the largest of the existing Sulawesian endemics, were distinctly rare to absent in overlying deposits, where the only large animal represented in significant quantities is *S*. *celebensis*.

**Fig 9:**
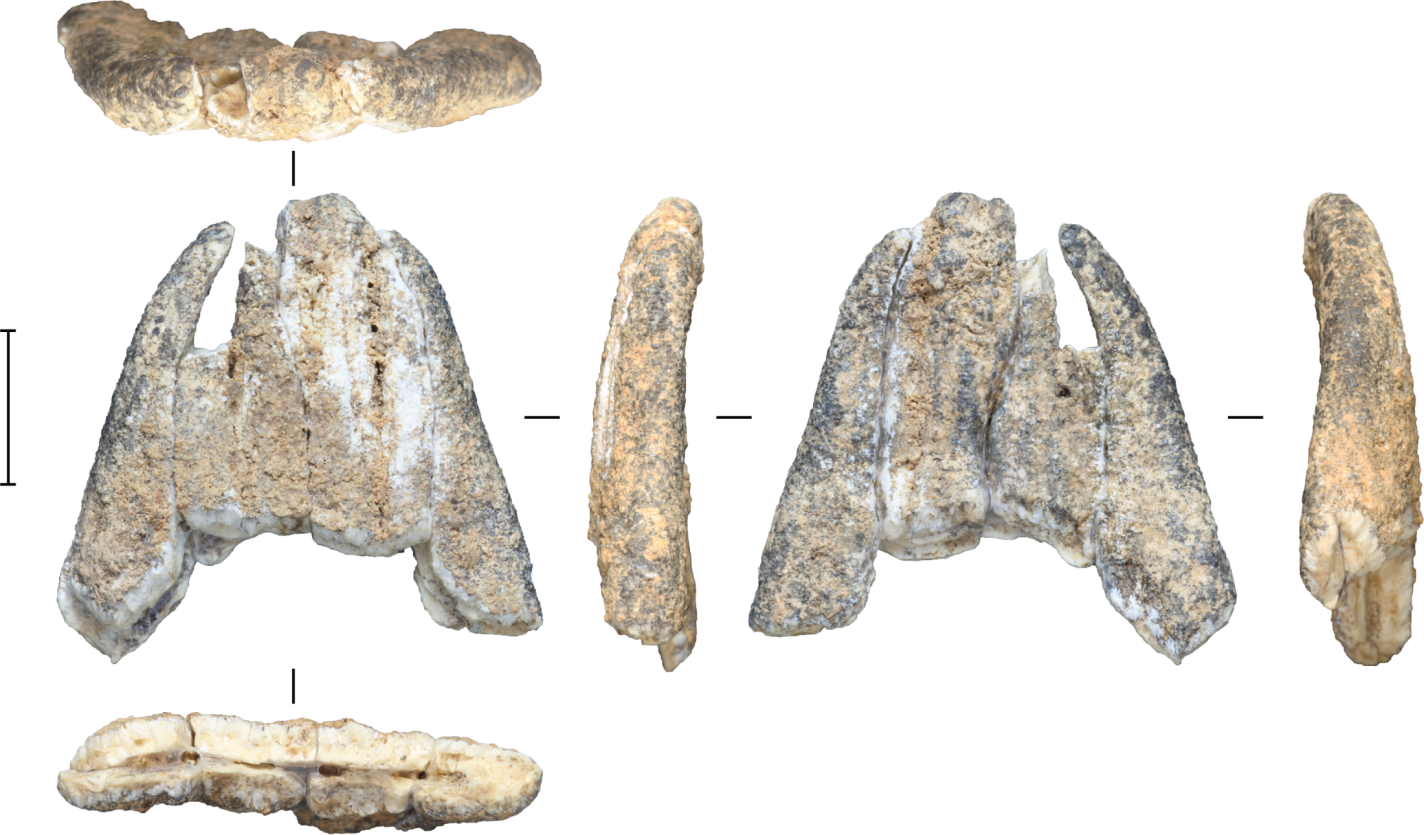
Elephantinae (genus and species indeterminate) molar plate fragment excavated from Layer I at Leang Burung 2. Scale bar is 10 mm.

Importantly, excavations in Layer I also yielded the first evidence for the presence of extinct ‘megafauna’ at Leang Burung 2. A number of proboscidean molar fragments were recovered *in situ* below Layer V in square B10 (spits 18–19). The Layer I specimens represent six molar plate fragments, and a single plate (Fig 9), all from milk molars, judging from the limited enamel thickness (1.4–2.5 mm). The enamel is double layered, with the inner enamel layer about two-thirds or more of the total enamel thickness. In longitudinal cross-section, the plates were clearly not wedge-shaped, and this characteristic, combined with the relatively thin outer enamel layer, suggests that the milk molars belong to a member of the genus *Elephas* or *Palaeoloxodon*, and not to *Stegodon*. The height of the largest fragment amounts to 32 mm, giving an indication of the minimum crown height. The base of the single plate is not preserved, so the degree of hypsodonty could not be established. To our knowledge, the proboscidean molar fragments from Layer I represent the only published evidence available thus far for the presence of proboscidean fauna in association with stone tools and other human occupation remains in an excavated cave or rock-shelter deposit on Sulawesi.

Below, we report the results of our efforts to obtain a reliable chronology for the Layer I cultural horizon and associated findings.

### Evidence for human occupation below Layer I

**Fig 10:**
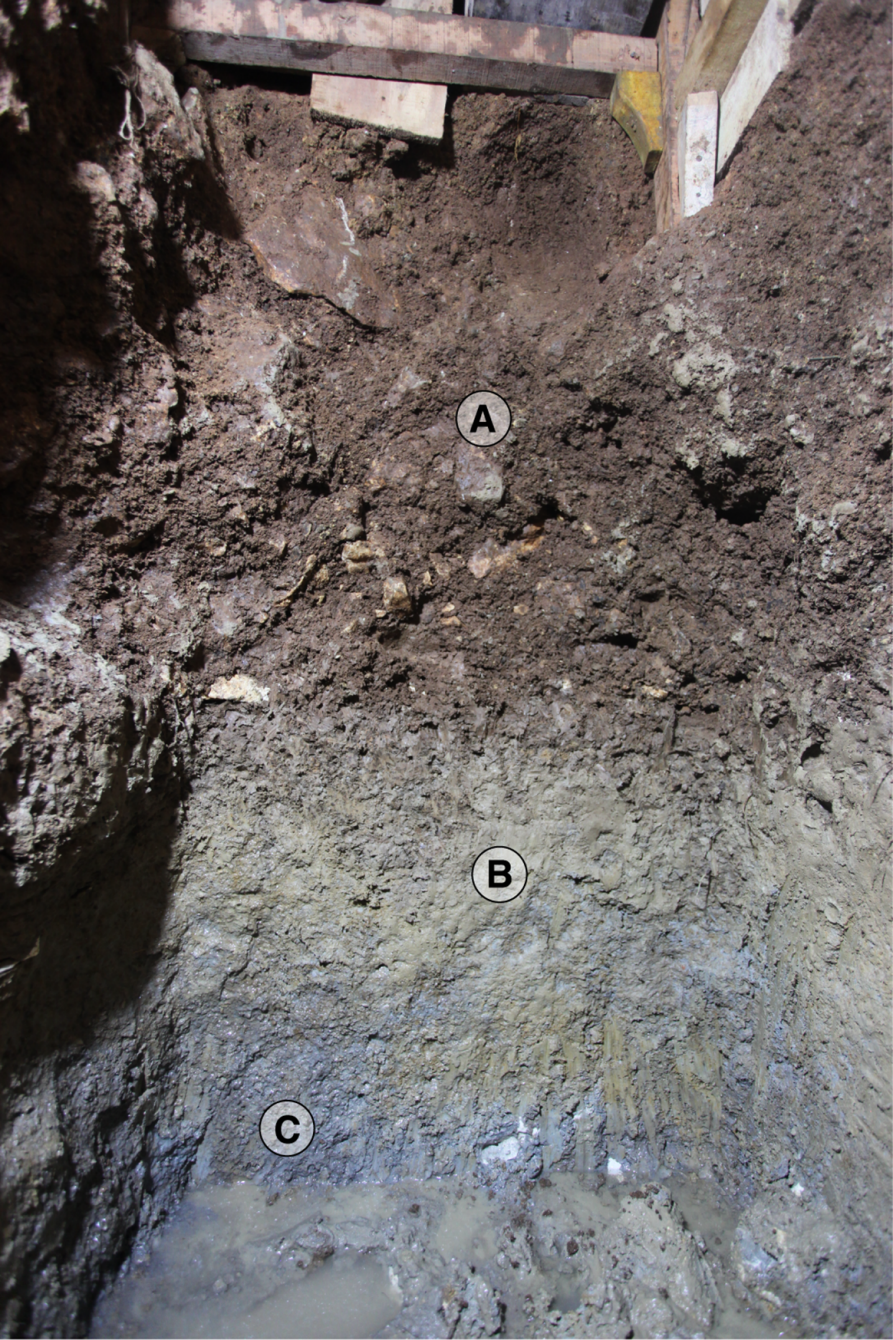
**The deep deposits at Leang Burung 2 (Layers A to C).** The photograph, taken during the 2011 excavations, is of the north wall of square D10 and the section covers a depth of approximately 5 m to 6.2 m below the surface.

**Fig 11:**
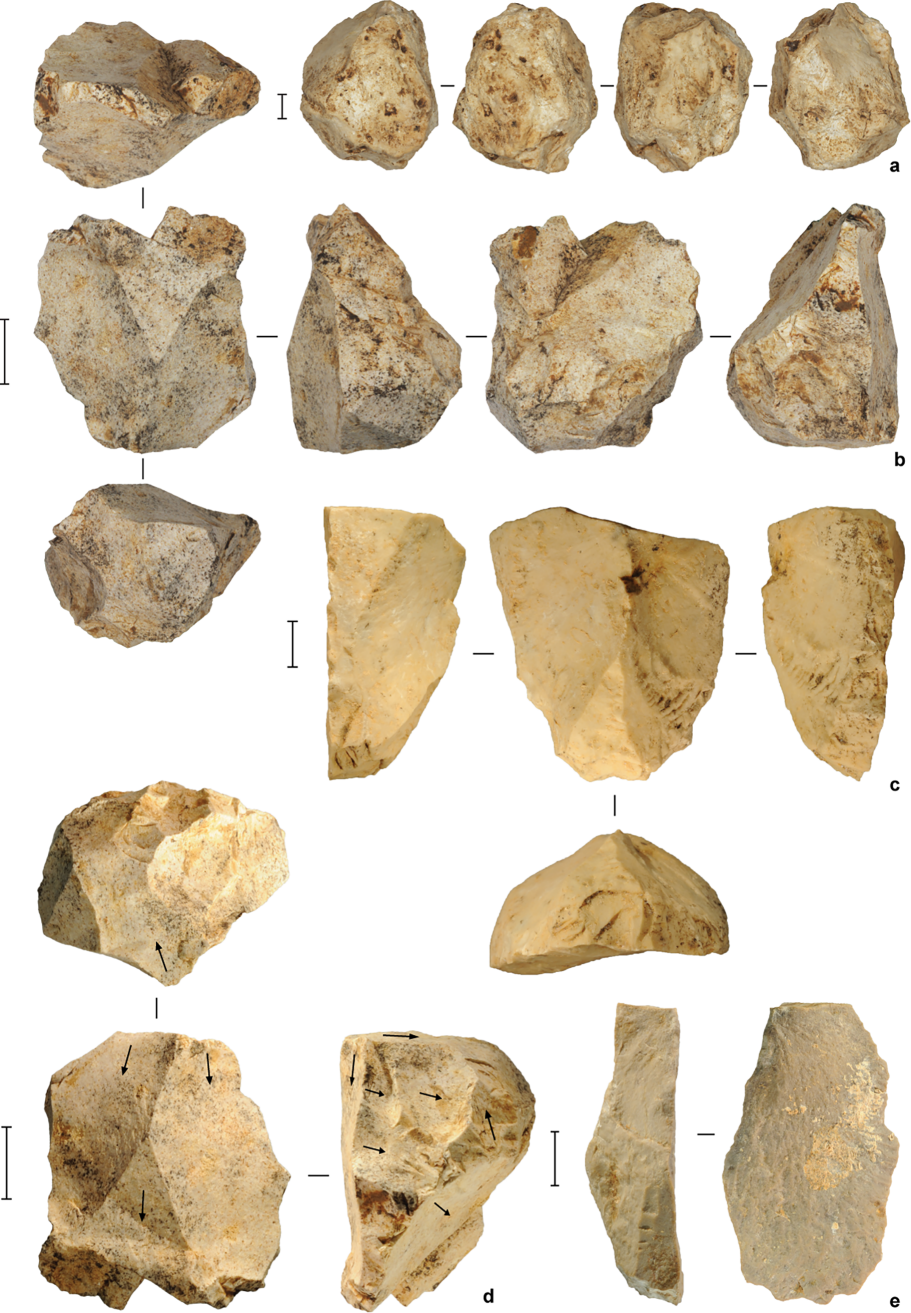
Stone artifacts from the deep deposits at Leang Burung 2. (**a**) limestone core, square D10, spit 54 (Layer A); (**b**) limestone core, square D11, spit 55 (Layer A/B); (**c**) retouched limestone flake, square D11, spit 47 (Layer I); (**d**) multiplatform limestone core, square D11, spit 55 (Layer A); (**e**) limestone flake, square D11, spit 50 (Layer A). Scale bars are 10 mm.

**Fig 12:**
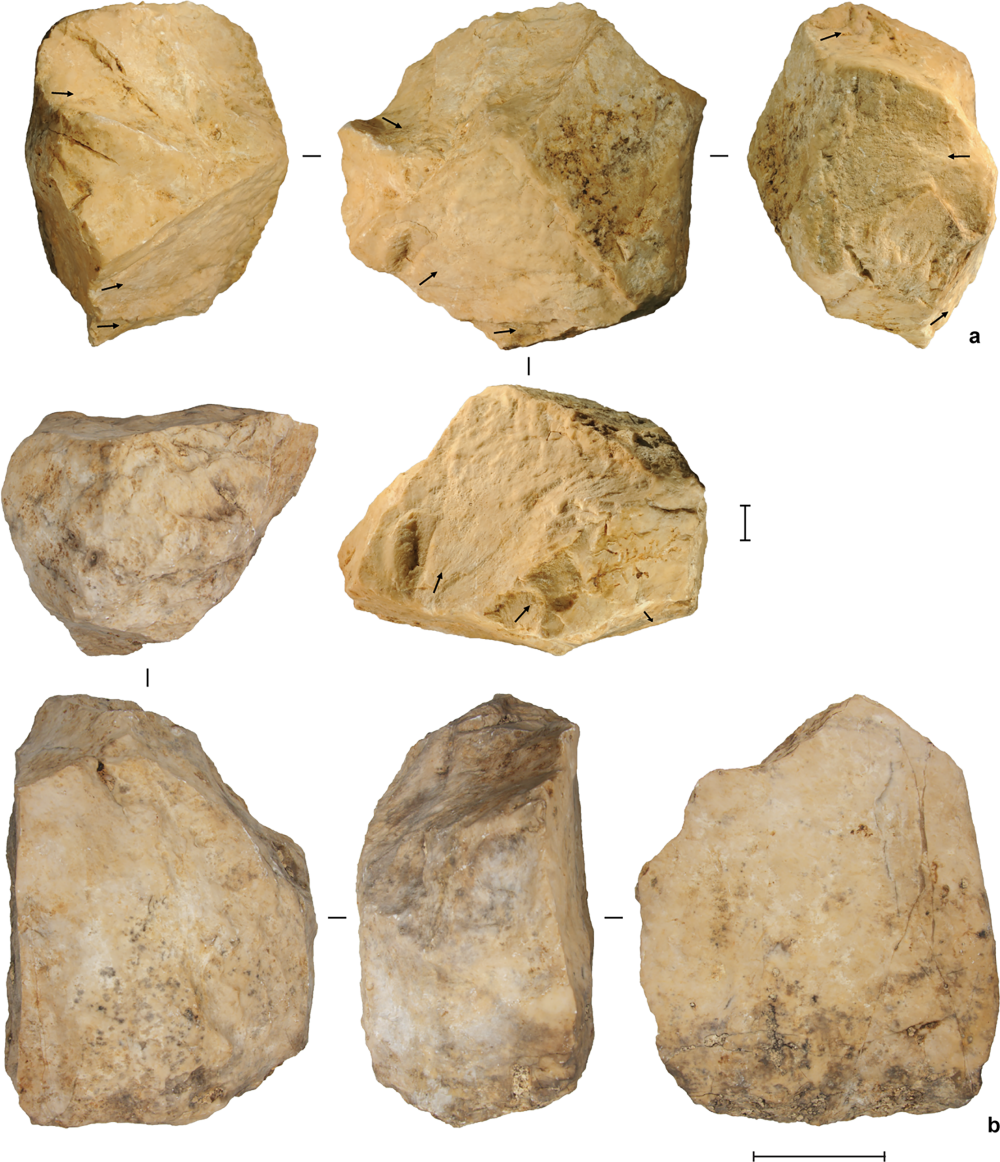
Stone artifacts from the deep deposits at Leang Burung 2. (**a**) limestone radial core, square D10, spit 52 (Layer A); (**b**) large single platform limestone core, square D10, spit 52 (Layer A). Scale bars: **a**, 10 mm, **b**, 50 mm.

**Fig 13:**
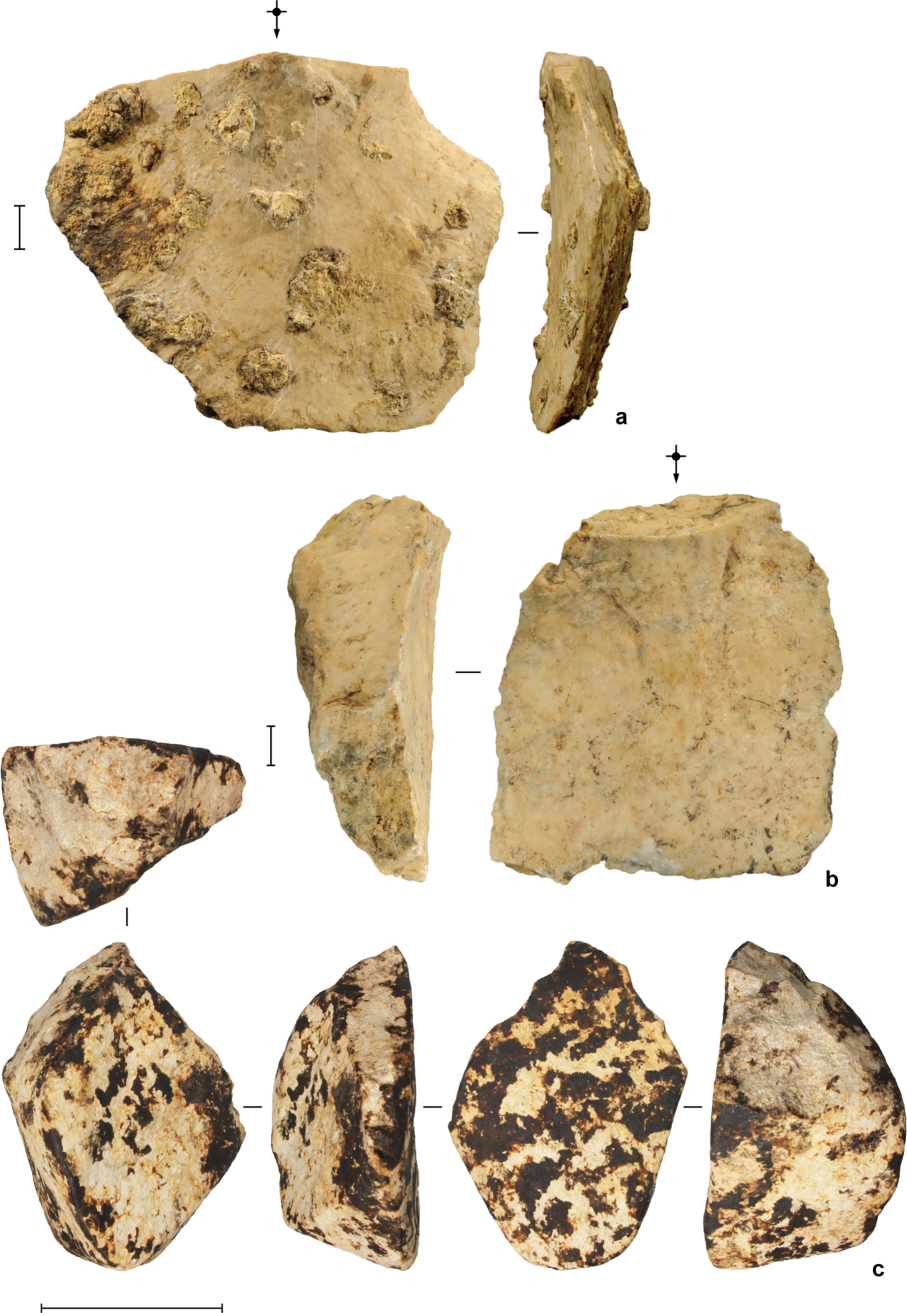
Stone artifacts from the deep deposits at Leang Burung 2. (**a**) limestone flake, square D10, spit 51 (Layer A); (**b**) limestone flake, square D11, spit 48 (Layer A); (**c**) unifacially retouched limestone cobble ‘pick’, square D10, spit 55 (Layer A). Scale bars: **a**-**b**, 10 mm, **c**, 50 mm.

**Fig 14:**
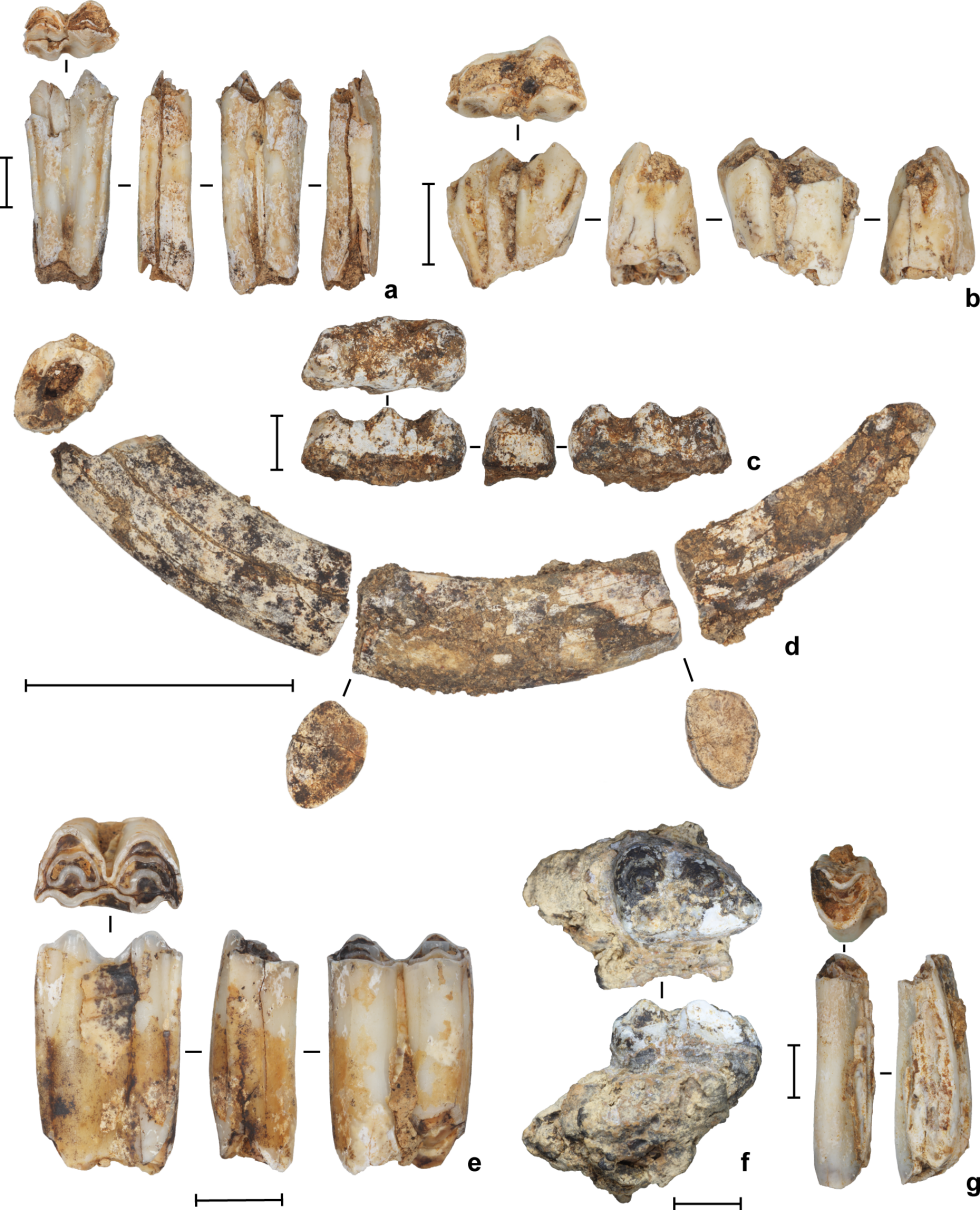
Fossil fauna from the deep deposits at Leang Burung 2. (**a**) Anoa (*Bubalus* sp.) molar, square D11, spit 45 (Layer I/A); (**b**) anoa molar, square D11, spit 50 (Layer A); (**c**) Suidae M3, square D11, spit 51 (Layer A); (**d**) babirusa (*Babyrousa* sp.) lower canine (Layer A); (**e**) anoa molar, square D11, spit 51 (Layer A); (**f**) Suidae molar, square D10, spit 51 (Layer A)–NB: this tooth is U-series sample #3011A/B, which yielded a minimum age of 113 ± 10 ka (see Table 3); (**g**) anoa molar fragment, square D11, spit 60 (Layer B). Scale bars: **a**-**c**, **e**-**g**, 10 mm, **d**, 50 mm.

The 2011–13 excavations at Leang Burung 2 resulted in the discovery, beneath Layer I, at a depth of about 4.5 m to 5 m below the surface, of a sedimentary horizon that had not been encountered during the 1975 and 2007 excavations (Figs 6 and 10). This newly identified deposit is up to 90 cm in thickness and comprises a dark brown sandy soil (Layer A) with abundant angular limestone roof-fall fragments. Excavations in Layer A yielded 250 flaked stone artifacts (Figs 11–13) and a large faunal assemblage dominated by fossil remains of rodents (most of which probably reflect natural biota), but with relatively high proportions of diagnostic remains of anoas, and large quantities of small unidentifiable bone fragments belonging to large-bodied vertebrate mammals (e.g., anoas, *Sus* sp. and babirusa; Fig 14). Three refitting fragments of a 15 cm-long *Babyrousa* sp. lower canine were recovered (Fig 14D). The deposit contained no *in situ* shell. Most of the faunal elements in Layer A were heavily mineralized. Although there is no sharp boundary between Layer I and Layer A, there is a discernable difference in terms of the increase in the amount of sticky clay in Layer A.

The stone-flaking technology evident in Layer A is markedly different from the distinctive, chert-based technology documented by Glover [1], and by us, higher in the sequence in Layers V, IV, II and III/IIIa-b, which post-dates 35 ka cal BP. The Layer A artifacts reflect a highly expedient approach to lithic reduction. Local limestone cobbles were knapped by hard-hammer percussion. The resultant cores and flaked cobbles are generally large in size, measuring up to 16 cm long and weighing up to 3.5 kg (average maximum dimension = 85.8 ± 36.4 mm), and minimally reduced, with an average of 7 ± 4 flake removals. One cobble has two unifacially retouched sides that converge to form a ‘pick’-like projection (Fig 13C). Some large flakes (N = 3) were irregularly retouched. In terms of other stone tool assemblages from Sulawesi, the Layer A artifacts are most similar to the late Middle Pleistocene implements from Talepu [10]. They are also strongly reminiscent of patinated, water-rolled stone tools of the ‘Cabenge’ industry, which occur alongside fossils of *Stegodon* and other extinct endemics on the surfaces of open-sites in the Walanae Depression [56] (see also [57, 58]). For example, the large unifacially flaked pick-like tool from Layer A at Leang Burung 2 is comparable in form to a Cabenge pointed uniface [57, 58].

Below Layer A, cultural deposits continued in the form of small numbers of *in situ* limestone artifacts, and relatively plentiful vertebrate faunal remains (Fig 14G), in a horizontally-inclined, brownish grey to reddish brown silty clay (Layer B) (Fig 6). Underlying this oxidized pond deposit is a flat-lying, blue-grey to olive silty clay (Layer C) containing occasional vertebrate fossils, mostly from rodents and rodent-sized animals, but including some diagnostic anoa fossils. The latter deposit occurred right to the base of the 2011 trench (6.2 m depth in square D10), at which point excavations were discontinued due to groundwater intrusion. Teeth from extinct juvenile sharks and other tropical inshore marine species were recovered from Layers B-C. These fossils had most likely eroded from the limestone bedrock of the Tonasa Formation (J.CA. Joordens, pers. comm., 2015).

### Chronology of Layer I and underlying deposits

Our attempts to establish a chronology for Layer I and the newly identified strata below (Layers A-C) have relied on luminescence dating of sediments, U-series analysis of faunal remains, and U-series analysis of straw stalactites, owing to the lack of charcoal, shells, and other organic matter within these contexts. Concerning the latter, little nitrogen (<0.1%) was preserved in the dentine from a sample of teeth found in Layer I (n = 1), Layer A (n = 4), and Layer B (N = 5), suggesting that there was no collagen present in these specimens [59].

If deposition of Layer I (and any underlying strata) post-dated the erosion of the shell-rich breccias, then our excavations in these layers should have recovered consistent evidence for the presence of *in situ* shells, chert artifacts, and other findings characteristic of the post 35 ka cal BP assemblages. This was not the case, however; indeed, strong vertical patterning in cultural and faunal assemblages means that the archaeological findings from Layer I and below are straightforward to distinguish from those recovered from Layers II and above. As already noted, the chert-based lithic technology (and the use of chert as a raw material) is restricted to the upper levels (Layers V, IV and II), whereas the lower levels (Layers I and A) contain a distinct, Talepu-like technology that predominately involves the use of dense limestone as raw lithic material. Moreover, diagnostic remains of anoas and babirusas are generally confined to the lower levels (Layers I and below). The concentration of vertebrate faunal remains at the base of the excavated sequence (Layers A-C) is much higher than in the upper levels excavated by us (i.e., Glover’s Layers V, IV, II, I and III/IIIa-b). For instance, the Minimum Number of Individual Specimens (NISP) reaches up to 350 specimens per spit in the lower, newly identified units, whereas in the overlying strata it reaches a maximum of only 80 specimens per spit (spit 46, 450–460 cm below the surface, Layer II).

Fossilization also appears to be different in Layers A-C. The bone of fossil fragments from these lower levels is often poorly preserved, multicolored (white, beige, grey and brown), and exhibits black superficial staining (probably manganese coatings), variable degrees of abrasion, and, almost invariably, dissolution pitting. Many skeletal elements demonstrate hairline cracking with exfoliation of cortical bone surfaces. Almost all the fragments from the lower levels are partially or entirely covered with yellow-brown calcrete. Calcrete precipitation has further damaged the bone by causing cracks. In the upper levels (Glover’s Layers V, IV, II, I and III/IIIa-b), bone preservation is generally much better, although breakage and fragmentation is comparable with lower levels. Cortical bone exfoliation occurs, but strong dissolution pitting is less frequent. Instead of irregular calcrete attachments, bones from the upper levels are often entirely covered with a thick encrustation of brownish-grey calcite. The bone typically has an even coloration (i.e., white, dark brown, bluish grey, grey, or salmon in hue), except when it has been burnt. Unlike specimens in Layers A-C, which display no indications of exposure to heat, bone from the upper levels frequently (~15%) shows clear signs of burning, as evidenced by polygonal hairline cracks and charred, black coloration. Some burnt bones from Layers II-V show sharp color transitions in cross-section, the inner part being evenly grey colored and the outer zone white and calcined. Other specimens are entirely calcined, leaving a white, porcelain-like texture.

The bone and tooth samples sectioned for U-series dating (see below) also exhibit distinct internal coloring corresponding to their specific depositional context. Fossils from Layer I and A display a white internal color, whereas those from Layer II have a darker, chocolate-coloured internal bone structure. This suggests distinct burial histories for these fossils, adding further weight to the notion that there is minimal evidence for mixing across the main upper and lower stratigraphic levels identified thus far at Leang Burung 2.

Given these observations, we argue that Layers I and A-C must have been deposited prior to the formation and subsequent erosion of the overlying shell-rich strata that are now represented only by the remnant, cemented breccias on the rear wall. We can therefore nominally infer a minimum age of at least ~35 ka cal BP for uppermost Layer I.

The 2007 investigations involved the first attempt to establish a luminescence chronology for Layer I. Owing to the limitation of the radiocarbon barrier at ~40 ka, and the nature of the quartz in this volcanic province, the red thermoluminescence (red TL) dating technique was used (see Methods). Red TL dating of volcanic quartz grains from Layer I (Fig 6) yielded three Late Pleistocene age estimates (Table 5), including a possible age of ~82 ka for Layer I at a depth of 4.4 m. Due to limitations of the red TL technique, these results should be regarded as maximum ages only for sediment deposition. It should also be noted that the three red TL age estimates all overlap at the 2-sigma (σ) interval, or 95% probability.

**Table 5:** Results of red TL dating of mineral grains from Leang Burung 2 (2007 field season).

Sample	Sample	Field gamma	Beta	Cosmic-ray	Water	Total	Signal^f^	Equivalent	Age
code^a^	Depth	dose rate	dose rate	dose rate	content	dose rate		dose	
	(m)	(Gy kyr^-1^)^b^	(Gy kyr^-1^)^b^	(Gy kyr^-1^)^c^	(%)^d^	(Gy kyr^-1^)^e^		(Gy)^g^	(kyr)^h^
SLBG2-1	3.00	1.430 ± 0.022	1.465 ± 0.067	0.080 ± 0.008	20 / 15 ± 5	3.01 ± 0.18	Red TL U	317 ± 17	106 ± 9
							Red TL B	121 ± 36	40 ± 12
SLBG2-2	3.60	1.430 ± 0.022	1.205 ± 0.067	0.078 ± 0.008	20 / 15 ± 5	2.75 ± 0.16	Red TL U	339 ± 17	123 ± 10
							Red TL B	208 ± 44	76 ± 17
SLBG2-3	4.40	1.430 ± 0.022	1.554 ± 0.059	0.075 ± 0.007	18 / 12 ± 4	3.09 ± 0.15	Red TL U	336 ± 17	119 ± 8
							Red TL B	252 ± 63	82 ± 21

a Samples processed using the 90–125 μm size fraction.

b U, Th and K concentrations measured using a portable gamma-ray spectrometer at field water content to estimate the field gamma dose rate

combined with beta counting measurements of dried and powdered sediment samples in the laboratory.

c Time-averaged cosmic-ray dose rates (for dry samples), each assigned an uncertainty of ± 10%.

d Field/time-averaged water contents, expressed as (mass of water/mass of dry sample) x 100. The latter values were used to calculate the total dose rates and ages

e Mean ± total (1σ) uncertainty, calculated as the quadratic sum of the random and systematic uncertainties.

f Red TL = red thermoluminescence—U, unbleachable signal (i.e., light-insensitive signal, last zeroed when the grains were heated), B, bleachable signal (i.e., light-sensitive signal, last reset when the grains were exposed to sunlight).

g Equivalent doses include a ± 2% systematic uncertainty associated with laboratory beta-source calibrations.

h Uncertainties at 68% confidence interval.

Subsequently, the pIRIR_290_ dating procedure was conducted on sediment samples collected in 2012 from Layer B (LB2-OSL13), Layer A (LB2-OSL12 & LB2-OSL11), and Layer I (LB2-OSL10 & LB2-OSL6) (see Methods). Samples LB2-OSL6 and LB2-OSL10 from Layer I yielded internally consistent ages of ~45–35 ka and have lower age limits at 2σ of ~27–25 ka (Table 2). Significant age inversion was seen for the other sample from Layer A (LB2-OSL12), and from the lowermost sample (LB2-OSL13), which was collected from underlying Layer B. These age inversions are likely due to very high levels of uranium (>10 ppm) towards the base of the trench, as reflected by the abnormally high environmental dose rates for these two samples compared with the others. For example, the dose rate dramatically increases from ~2.6 Gy/ka for sample LB12-OSL11 to ~4.0 Gy/ka and ~5.4 Gy/ka for LB12-OSL12 and LB12-OSL13, respectively. Given the similar D_e_ values for these samples, it is possible that the high environmental dose rates for the deepest samples are affected by recent uptake of U. Laser ablation U-series analysis of fossils suggests a complex history of U mobilization in buried faunal remains (see below). Hence, anomalies in the ages from the two samples LB2-OSL12 and LB2-OSL13 could be a consequence of the presence of a fluctuating, U-enriched groundwater table, which has complicated our efforts to accurately and reliably estimate external dose rates for the deposit.

If a recent uptake of U from groundwater for the two samples (LB2-OSL12 and–OSL13) is assumed, and we use the weighted mean dose rate (2.77 ± 0.22 Gy/ka) for those from the upper samples as ‘effective dose rates’, the ‘dose-rate corrected’ ages for the LB2-OSL12 and LB2-OSL13 are 51 ± 6 and 44 ± 5 ka, respectively, which are statistically indistinguishable from each other and the age of LB2-OSL11. We therefore argue that the ‘dose-rate corrected’ ages are more reliable and can be tentatively taken to estimate the ages of the two lowermost samples (LB2-OSL12 and LB2-OSL13). However, further confirmation of these age estimates is needed in order to model the open-system geochemistry that may be confounding the results of luminescence dating. Acquiring more information on the recent hydrology at Leang Burung 2 may also help to clarify matters, as may carrying out further experimental work that is beyond the scope of the present study.

We also conducted laser ablation U-series analyses on a total of 11 fossil specimens excavated in 2011 and 2012 from Layer I (n = 7) and Layer A (n = 4). The resultant data are shown in Table 3. The dated specimens are all at least early Holocene to Late Pleistocene in age. Aside from one specimen from Layer A (sample #3011A-B), which we will discuss below, the oldest minimum age for sampled faunal remains in the deposit is ~17 ka (Layer I). It should be recalled that teeth and bone are open systems, and thus, the U/Th measurements can only provide minimum age estimates for these specimens [60]. The proboscidean molar fragment from Layer I yielded a minimum U-series age of 8.1 ± 0.2 ka, a result that is very likely to underestimate its true age, and that of the most recent appearance of endemic proboscidean fauna on Sulawesi, by a significant margin.

It seems plausible to argue that the predominately young minimum U-series ages for the faunal remains buried at Leang Burung 2 reflect climatic changes in the post-LGM period. High-resolution palaeoclimate records derived from lake sediment cores in central Sulawesi indicate a very wet climate and closed-canopy rainforest during much of MIS3 (~58 to 30 ka; [61, 62]). This was followed by the abrupt onset of a period of severe drying (~33–16 ka) in the transition to the LGM [61]. Biomarker records (δ^13^C_WAX_) from lake sediments suggest grassland and open-canopy forest expanded during a more arid and seasonal MIS2. A return to wetter and more humid conditions is evident by ~15 ka during the transition to the interglacial, with the δ^13^C_WAX_ reaching its Holocene average at around 11 ka [61]. There is evidence for a major increase in regional rainfall by 12–10 ka (e.g., [61]). In sum, we propose that the relatively young minimum ages for faunal remains at Leang Burung 2 may reflect greater U solubility and mobilisation during times of increased precipitation following the LGM, perhaps owing to a rise in the U-enriched groundwater table at the site.

Only one fossil in the dated assemblage, a suid molar from Layer A (sample #3011A-B), appears to preserve the isotopic signature of pre-LGM U-uptake (Table 3). The best age estimate for this sample comes from averaging the isotopic data from the dentine and attributing the variations to micro-migration. Utilizing the DAD diffusion model [63], the minimum age for this tooth would be ~100–80 ka. This suid molar was encased within a thick covering of indurated calcrete that appears to have shielded the specimen from further uptake (or loss) of U. Although this fossil has a minimum age of 100–80 ka, the U^234^/U^238^ ratio for it differs markedly from that of other U-series dated fossils within Layer A (Table 3). This suggests that this suid molar was probably reworked from deeper within the deposit.

We also carried out solution U-series dating on a broken straw stalactite recovered *in situ* 520–530 cm below the surface in Layer A (n = 1), yielding a U-series age of between 49.6–45.8 ka (Table 4; sample name: STRAW-08-R1/R2)–in this case, the ‘tip’ end of the soda straw was slightly younger than the opposite end (the ‘base’), confirming the reliability of the U-series dates. The U-series age for this straw stalactite now provides a maximum age of ~50–46 ka for Layer A. This age estimate is consistent with the older age limit of the fading corrected pIRIR age for Layer A (LB2-OSL11), which at 2σ is ~44 ka (see above). It is also consistent with the argument that deposition of the lower levels (Layers A-C, and also overlying Layer I) pre-dates the earliest formation of the above-noted unconsolidated archaeological deposits that survive now only as remnants in the high-level breccias.

**Fig 15:**
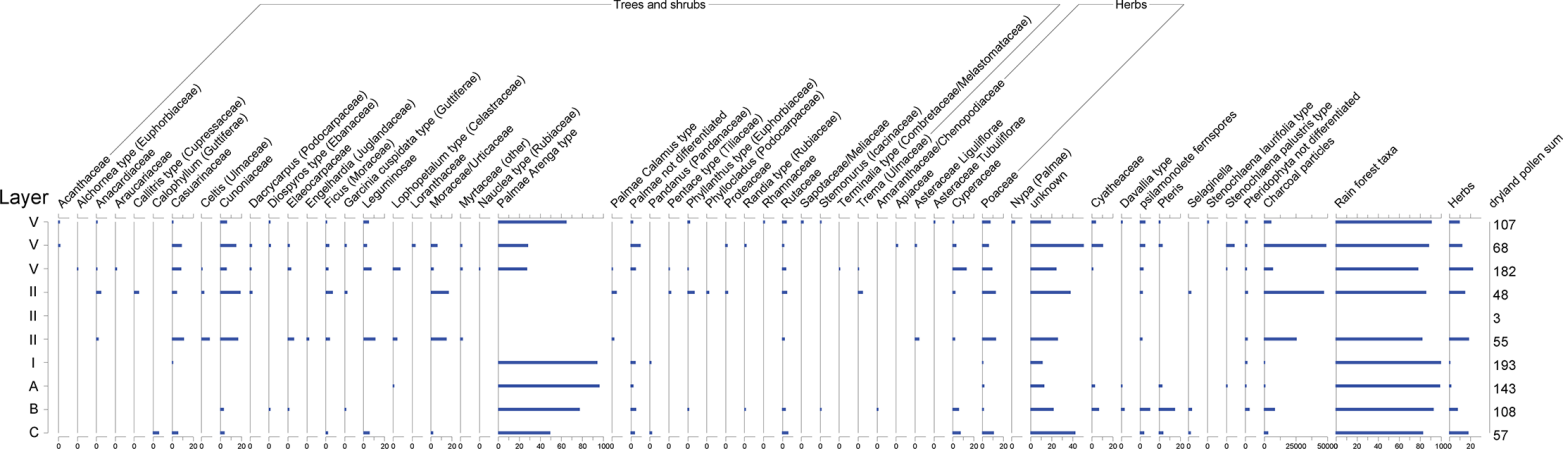
Results of Leang Burung 2 pollen analysis.

Finally, we conducted pollen analyses on a total of 10 sediment samples collected from the lower (Layers I and A-C) and upper levels (Layers II and V) (Fig 15). Pollen preservation was generally good and did not vary stratigraphically. The pollen assemblages suggest the continuing presence of forest vegetation (dominated by *Arenga* sp.) around the site during the deposition of these units, with only a minor contribution from herbs and ferns. The latter likely indicates that the forest was a type of rain-green or monsoonal lowland forest. The pollen assemblages from Leang Burung 2 are consistent with the view that Layers I and A (and possibly B-C) were deposited during wet and humid conditions of MIS3, whereas formation of the upper sequence (Layers II-V) is a Holocene phenomenon.

## Discussion

In this paper, we have reported the preliminary results of our recent archaeological excavations (2007 and 2011–2013) at Leang Burung 2 rock-shelter. A key finding is that the upper part of the sedimentary sequence has been affected by a major and previously unrecognized erosion and re-infilling event that casts doubt on the reliability of Glover’s model of human occupation at the site 35–24 ka cal BP. We have now identified the base of Layer I below the deepest level reached by Glover [1] in 1975, but this deposit has thus far provided few opportunities for reliable dating. We also recovered evidence for a human presence in stratified and undisturbed layers below Layer I. These previously undocumented cultural horizons occur below the level of the disturbance zone affecting the upper strata, and artifacts and faunal remains from these deposits differ strikingly from those post-dating 35 ka cal BP. As with Layer I, however, we have only succeeded in establishing a coarse-grained chronology for these basal cultural levels. Despite these problems, our excavations well below the lowest point exposed by Glover in 1975 have documented evidence for a long sequence of cultural deposits at Leang Burung 2, including hitherto unknown and enigmatic indications of a human presence in deep trenches that still have not reached bedrock.

One of Glover’s [1] most significant finding in 1975 was the identification of a purportedly Late Pleistocene modern human occupation sequence spanning ~35 to 24 ka cal BP, for which artifactual evidence was gleaned from the upper levels of the site (Layer II and overlying strata, Layers IV and V). Our own excavations conducted at this shelter between 2011 and 2013 raise serious doubts about the proposed antiquity of the sedimentary deposits that yielded evidence for this cultural phase (hereafter, the ‘Glover complex’). Constructing an accurate and reliable chronological framework for the site has proved challenging. However, we have now accrued sufficient evidence to propose the following model–which is invariably provisional–for the complex depositional history of this rock-shelter site.

As reported above, ^14^C-dating of freshwater gastropod shells collected from flowstone-capped breccias perched on the rear wall of the shelter above the present-day ground floor surface suggests that a series of intact occupation strata falling within the time range of ~35 to 23 ka cal BP once existed at this site, but have been almost completely removed by erosion. The cemented breccias visible at various places on the rear wall of the shelter seem to comprise the only vestiges of this pre-LGM sedimentary sequence. The majority of shells excavated from Layers II, IV and V, and certainly those employed by Glover [1] to establish a chronology for these strata, in our view, more than likely eroded out of the ancient midden-bearing breccias and become incorporated into these younger accumulating deposits, along with stone artifacts, bones, and other archaeological materials.

It would appear, therefore, that the ‘Glover complex’ consists of archaeological materials of mixed ages that were deposited long after the LGM. We can infer that the ‘gutting out’ of the original sequence of unconsolidated, pre-LGM floor deposits occurred during the terminal Pleistocene and that subsequent re-infilling of a deep, channel-like erosional feature in Layer I may have taken place during the early Holocene (~10–9 ka), as suggested by straw stalactite U-series ages (Table 4). It follows that the key archaeological components of the ‘Glover complex’, unifacial macroblades with Levallois-like characteristics, and silica gloss residues, as well as lithic reduction methods attributed to this phase generally, cannot now be conclusively assigned to the Late Pleistocene period.

However, it seems possible that not all of the archaeological materials within the upper ‘infill’ deposits simply eroded out of the pre-LGM breccias. For instance, with minimal effort we were able to identify 15 separate lithic conjoin sets in these strata from the assemblage excavated in 2011; in most cases (86.7%), refits were separated by vertical depths of <10 cm, with the maximum vertical distance between refits being ~21 cm. It is possible that refitting artifacts eroded out of the pre-LGM breccias and were deposited close to each other within mixed-age assemblages. It is also plausible, however, that humans were present at the site during the early Holocene when Layers II, IV and V were accumulating. If so, it is likely that these deposits formed before the emergence or initial local manifestation of the Toalean, as the characteristic implements of this Holocene industry (e.g., microliths and Maros points) [34] have never been found at Leang Burung 2, despite its proximity to a comparatively rich Toalean occupation site at nearby Leang Burung 1 [34, 35]. Previous research has yielded no evidence for a human presence in the Maros karsts between the LGM and middle Holocene [21, 64].

Such a scenario would account for the presence of a shell in Layer II dated to 9–8.6 ka cal BP (OZO824), and one from the lower levels dated to 9.6–9.5 ka cal BP (Wk33233) (Table 1). Both shells are very close in age to the five U-series-dated straw stalactites from Layer II, which range between ~10.7 and 9 ka (Table 4). These two early Holocene shells are most plausibly interpreted as intrusive cultural materials that became dislodged from the loose and unconsolidated sediments exposed in section in the upper trench walls during the 2011 excavations. The presence of these erroneously young objects in the deposit is consistent with humans being present at this site during the early Holocene, but apparently prior to the first appearance of the Toalean in this region.

Glover [1] interpreted the steeply sloping stratigraphy of Layers IV and II as reflecting subsidence and deformation of the deposit due to undermining by sinkholes from below. However, our deep-trench excavations revealed no traces of such features. At the base of the excavated section, Layers B and C comprise horizontally inclined oxidized pond deposits. These remnant landscape surfaces seem to reflect an early phase in the depositional history of the site when the shelter floor was periodically inundated by floodplain runoff from a nearby river. This was followed by lateral migration of the river and associated floodplain away from the tower base. Sediments derived from local sources then started to accumulate in the shelter, beginning with Layer A, which is also flat-lying, and then Layer I. Thereafter, Layers II, IV and V infilled a steep-sided depression in Layer I. It is for this reason, we contend, the latter deposits slope downwards from the rear of the shelter towards the front, not because they are formerly horizontally inclined deposits that are now slumping into sink holes or otherwise being warped and deformed by sink action, as had originally been proposed by Glover [1].

An important outcome of our excavations at Leang Burung 2 is the discovery of previously unknown cultural occupation levels that occur below the maximum depths attained by prior excavations in 1975. We verified Glover’s [1] observation that Layer I, a massive weathered soil horizon, contains cultural and faunal remains that contrast strongly with those in post-35 ka cal BP contexts. Furthermore, the 2011–13 excavations revealed that Layer I lies atop a ~90 cm thick silty clay (Layer A) from which we recovered stone artifacts and fossils from babirusas, anoas, and anoa-sized mammals. The strong bias in Layer A towards large-bodied forest-dwelling taxa is not due to differential preservation, as the recovery of many microvertebrate remains demonstrates. The poor surface condition of most fossils from Layer A precludes definitive assessment of the presence or absence of cut-marks and other signs of butchery. However, it seems unlikely that the abundance of ‘megafauna’ remains is due to carnivore accumulation, given that the largest non-human predators on Sulawesi were civets and pythons; moreover, other prolific accumulators of faunal assemblages on the Sunda Shelf, porcupines, are widely presumed to be Holocene introductions to the island fauna [23].

The cobble-based core-and-flake technology in Layer A is particularly enigmatic. This tool-kit is very similar to the technology at Talepu dated to ~194–118 ka [10]. The large flaked cobbles from Layer A closely resemble Cabenge core tools. Most Cabenge artifacts are from undated surface contexts. Previously thought to post-date the arrival of *H*. *sapiens* on Sulawesi [57, 58], recent findings [10] support earlier contentions [22] that at least some stone artifacts within these surface assemblages are late Middle Pleistocene in origin, and were perhaps made by an archaic hominin population [10].

Layer I and the newly revealed deposits underlying it lack materials suitable for ^14^C-dating, including charcoal, shell and bone collagen, and they have proved challenging to date using other methods. At best, based on pIRIR dating of feldspars from Layer A and U-series dating of a straw stalactite from this deposit, combined with the ^14^C chronology for the rear wall breccias, which are unequivocally younger from a stratigraphic perspective, we can constrain the time range of the deposits comprising Layers A to Layer I to between around 50 ka and 35 ka cal BP. The pIRIR age estimates for Layer I (~45–35 ka) are potentially consistent with the proposed timescale of the erosion and re-infilling episode, suggesting Layer I was deposited prior to accumulation of the ‘missing’ strata (~35–23 ka cal BP) represented now only by remnant breccias on the rear wall. Sample LB2-OSL11, from Layer A, yielded an earlier age of 73 ± 14 ka, although it is associated with a much larger uncertainty. The lower age limit for this sample at 2σ is ~44 ka. A suid tooth with a minimum U-series age of ~100–80 ka was also found in Layer A. Although this fossil could have been reworked, its presence signals the existence of faunal assemblages at the site that may extend back to MIS5e or earlier.

The identity of the hominins present at Leang Burung 2 during the early stages of this site’s formation history (i.e., Layers A, I and B) is unclear. The open site of Talepu 80 km northeast of Leang Burung 2 provides the first indication that Sulawesi harbours an early record of hominin occupation that very likely pre-dates the oldest known presence of our species in the region [10]. The excavated section at Talepu terminates at ~100 ka. Hence, the identity of the late Middle Pleistocene tool-makers, and what happened to them, are unresolved issues [10].

Recent findings show that modern humans were established in the lowland karsts of Maros by at least 40 ka, having brought with them an artistic culture focused on hand stencils and large figurative animal paintings [8]. These same inhabitants may also have been responsible for the cultural remains in Layers I and A. This possibility suggests that the first modern humans to colonize this region possessed a unique cultural repertoire that included selective exploitation of extinct forms of proboscideans and living ‘megafauna’, a cobble-based core tool technology comparable to that produced by late Middle Pleistocene hominins at Talepu, and one of the world’s earliest known rock art traditions. Alternatively, the original inhabitants of Leang Burung 2 may have belonged to an as-yet unknown population of archaic hominins that was later replaced by our species. If so, further research in the Maros-Pangkep karsts may help to determine not only the identity of these hominins but also whether or not there was a period of overlap (and perhaps interaction) with modern humans.

## Conclusion

Our 2007 and 2011–13 excavations at Leang Burung 2 have led to a revised understanding of the depositional sequence at this shelter site, a stalwart of regional prehistoric studies since the 1980s [1]. Contrary to previous understandings of this site, new dating evidence suggests the upper levels of the excavated floor deposits and the rear wall breccias perched above the present-day shelter floor actually represent two separate phases of deposition. Cultural and faunal remains in the stratigraphically deepest deposits at Leang Burung 2 (Layers I/A-C) pre-date the distinctively modern human culture identified at the site by Glover [1], but a more precise chronology for these lowermost levels, for the present time, is difficult to establish.

Archaeological excavations at Leang Burung 2 still have not revealed bedrock. Continued investigations at this site are likely to be severely hampered by the practical difficulties of excavating the deposit. In particular, extending the coverage, safely, to a stratigraphically deeper level than that attained by us will require dealing with the serious issue of groundwater intrusion, perhaps necessitating the use of a system of well points and continual pumping to lower the surrounding water table. The deep deposits were particularly hard to excavate from a technical perspective. However, assessing the time depth of the stratigraphic layers has proved the most vexing challenge. Leang Burung 2, like most limestone cave and rock-shelter deposits [20, 40, 65], has a complicated formation history.

Our research shows that there is a clear imperative to continue investigations in the lowland karsts of Maros-Pangkep. We concur with earlier assessments [20, 39] that many cave and rock-shelters in these karsts have been extensively disturbed and reworked by erosional processes and also by decades, perhaps centuries, of farmers stripping sites of mineral-rich earth [40]. Despite this, our deep-trench excavations at Leang Burung 2 have revealed that intact and relatively undisturbed occupation deposits of potentially great antiquity still exist at some sites in this lowland tower karst region. In our view, the Maros-Pangkep landscape holds enormous potential to illuminate the artistic culture and symbolism of Pleistocene human communities and the genesis of the Toalean, the enigmatic ‘Mesolithic’ tradition of Holocene South Sulawesi. Moreover, it is now apparent that further investigation may also reveal much earlier patterns of hominin settlement and faunal turnovers, including the extinction of proboscideans and other endemic species of ‘megafauna’, as well as, quite possibly, the late survival of archaic hominins and their eventual replacement by *H*. *sapiens*.

## Methods

### Radiocarbon dating

Freshwater gastropod shells were the only materials available for radiocarbon dating. Freshwater shells can be affected by a freshwater reservoir (or hardwater effect), which cause the shells to appear tens to thousands of years too old. Prior research in Maros highlights the complexities involved in acquiring accurate and reliable reservoir estimates for freshwater shells from this region (e.g. [21, 66]). As yet, there have been no attempts to conduct ^14^C-dating on the shells of *T*. *perfecta* and other freshwater gastropods collected live from karstic streams and rivers in the Maros region prior to the 1950s atmospheric testing of thermonuclear weapons [21, 67]. Accordingly, there are no widely accepted environmental offsets for correcting ^14^C ages on ancient freshwater shells [21]. Although we have treated the ^14^C dates on *T*. *perfecta* shells as relative ages, it seems reasonable to aver that they deviate from the true ages of the shells by hundreds, instead of many thousands, of years, although the latter possibility cannot be excluded. All ^14^C dates reported in this paper, including those of Glover [1] and the 2007 Leang Burung 2 excavation team, were calibrated using the Southern Hemisphere terrestrial calibration curve [49] and the OxCal 4.3 program.

### Red TL dating

**Fig 16:**
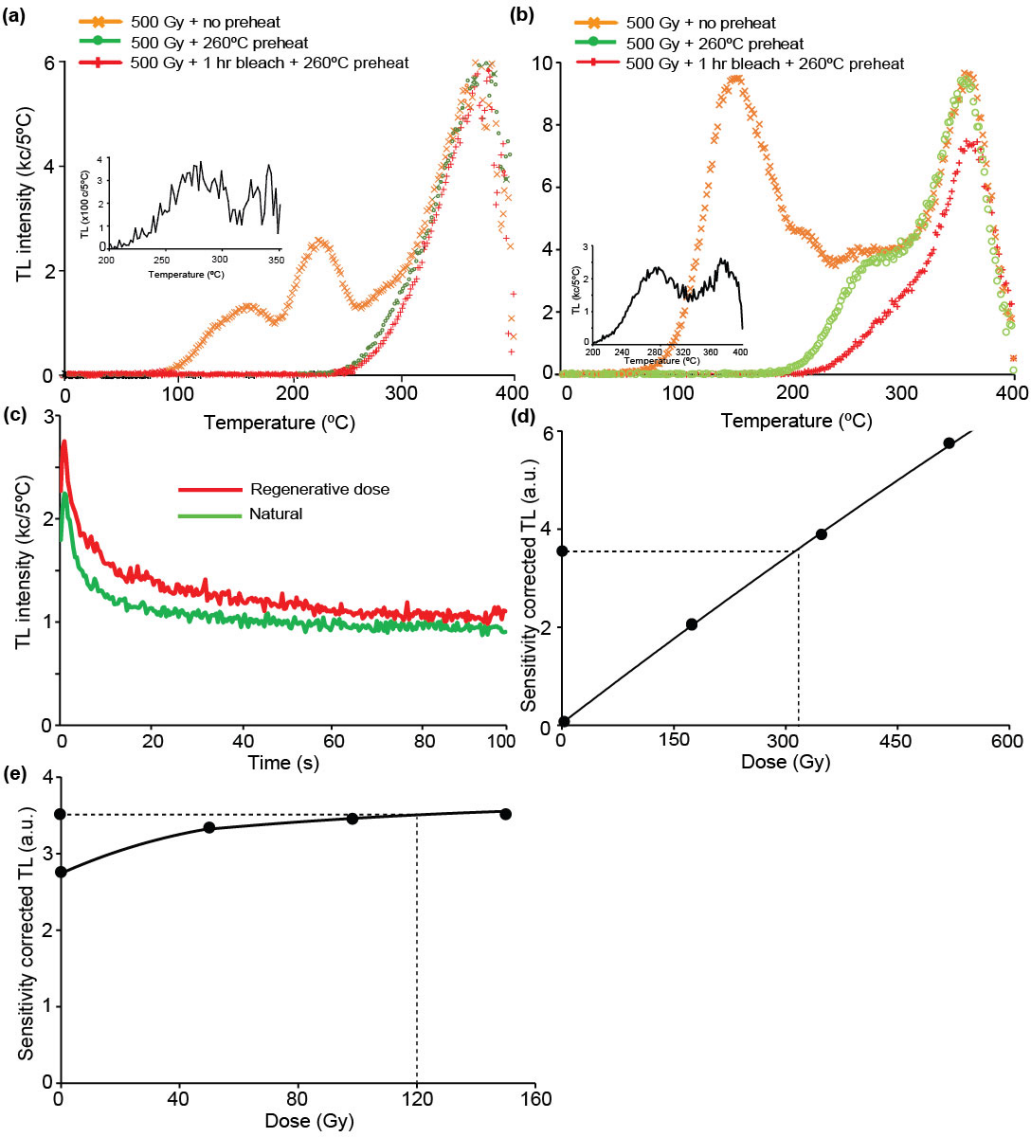
Example of the red TL data from Leang Burung 2. (**a**, **b**) a comparison of the red TL signal characteristics using glow curves derived from a Liang Bua cave (Flores) sample WR1 (a) and from a Leang Burung 2 sample (SLBRG2-1, Layer I). The glow curves demonstrate that after 500 Gy dosing the low temperature peaks disappear with the introduction of the 260°C preheat, and the presence of a light sensitive shoulder (260–305°C) that is removed by one hour of bleaching. The Leang Burung 2 sample shows similarities with the Liang Bua sample, but has a more defined bleachable shoulder and a more intense signal; (c) Isothermal decay of the red TL signal from sample SLBRG2-1; (d) dose response curve for the unbleachable signal derived from aliquot 1; and (e) the dose response of the bleachable signal isolated from data derived from aliquot 1 and 2 (see [70] for methodological details).

The 2007 investigations involved the first attempt to establish a luminescence chronology for Leang Burung 2. Owing to the limitation of the radiocarbon barrier at ~40 ka, and the nature of the quartz in this volcanic province, the red TL dating technique was used. Quartz grains of 90–125 μm collected from Layer I were separated using standard purification procedures, including a final etch in 40% hydrofluoric acid for 45 min [68]. Quartz grains were mounted on stainless-steel discs using silicone oil spray, each large aliquot being composed of ~5000 grains (~10 mg). The isothermal red TL emissions [69] were measured via a dual-aliquot protocol (DAP, [70]), using a red sensitive photomultiplier tube (Electron Tubes Ltd 9658B) and cooling tower (LCT50 liquid-cooled thermoelectric housing) with Kopp 2–63 and BG-39 filter combination, and laboratory irradiations were conducted using a calibrated ^90^Sr/^90^Y beta source. D_e_ were estimated from the 20–30 s interval of isothermal decay (which was bleachable by >380 nm illumination) using the final 160 s as background. Aliquots were heated to 260°C at a heating rate of 5 K s^-1^ and then held at 260°C for 1000 s to minimise the unwanted TL from incandescence. Palaeodoses were estimated from the 20–30 s interval of isothermal decay (which was bleachable by >380 nm illumination), using the final 160 s as background. Total dose rates were measured by *in situ* gamma spectrometry and laboratory beta counting. An effective internal alpha dose rate of 0.03 Gy ka^-1^ and a long-term water content of between 12–15 ± 5% were used. The red TL signal from the sediments at Liang Bua cave in western Flores has been extensively studied (e.g [65, 70–72]) and can be used for comparative purposes with the signal from the deposits in Leang Burung 2. Fig 16 displays this comparison, suggesting that the Leang Burung 2 sediments contain a similar signal intensity and wavelength of emission, and the same types of TL peaks and luminescence traps. These similarities provide confidence in the use of the DAP red TL technique as it was primarily developed for application to similar luminescence characteristics (e.g. [73]).

### pIRIR dating

Previous efforts to date sediments of volcanic origin from Indonesia have used red TL or ultraviolet OSL signals from quartz, and IRSL signals from potassium-rich feldspar (K feldspar). There is evidence to suggest that the OSL signals from these Indonesian materials are, in general, too dim for dating purposes [70, 74]. It can be argued, for instance, that the light-sensitive component of the red TL signal is less easily bleached than the OSL or IRSL traps, is small in size and is obtained by subtraction, possibly resulting in imprecise and potentially inflated ages [70]. The IRSL signals measured previously showed exceptionally high rates of anomalous fading and a suitable correction could not be applied. It was not until recently that the newly developed post-infrared infrared stimulated luminescence (pIRIR) [75] and multiple-elevated-temperature pIRIR (MET-pIRIR; [76]) procedures were successfully applied to date the sediments from this region, including at the open-air site of Talepu in Sulawesi [10], at the cave of Liang Bua on Flores [65], and at a recently excavated cave deposit at Leang Bulu Bettue 1.5 km to the north of Leang Burung 2 [77] (see also [64]).

Similar to the samples from the nearby site, Leang Bulu Bettue [77], the feldspar grains at Leang Burung 2 are relatively dim, so we could not apply the MET-pIRIR procedure. Instead, sufficient signal intensity can be obtained using a two-step pIRIR_290_ procedure [78], in which a IR bleach is given at a low temperature (e.g., ~50°C) before the pIRIR signal is measured at a high temperature (290°C). In this procedure, a preheat at 320°C for 60 s was applied for both the regenerative and test doses, and a hot-IR bleach at 340°C for 100 s was applied at the end of each regenerative and test dose measurement cycle (see [77], for details about the facility and experimental procedures).

**Fig 17:**
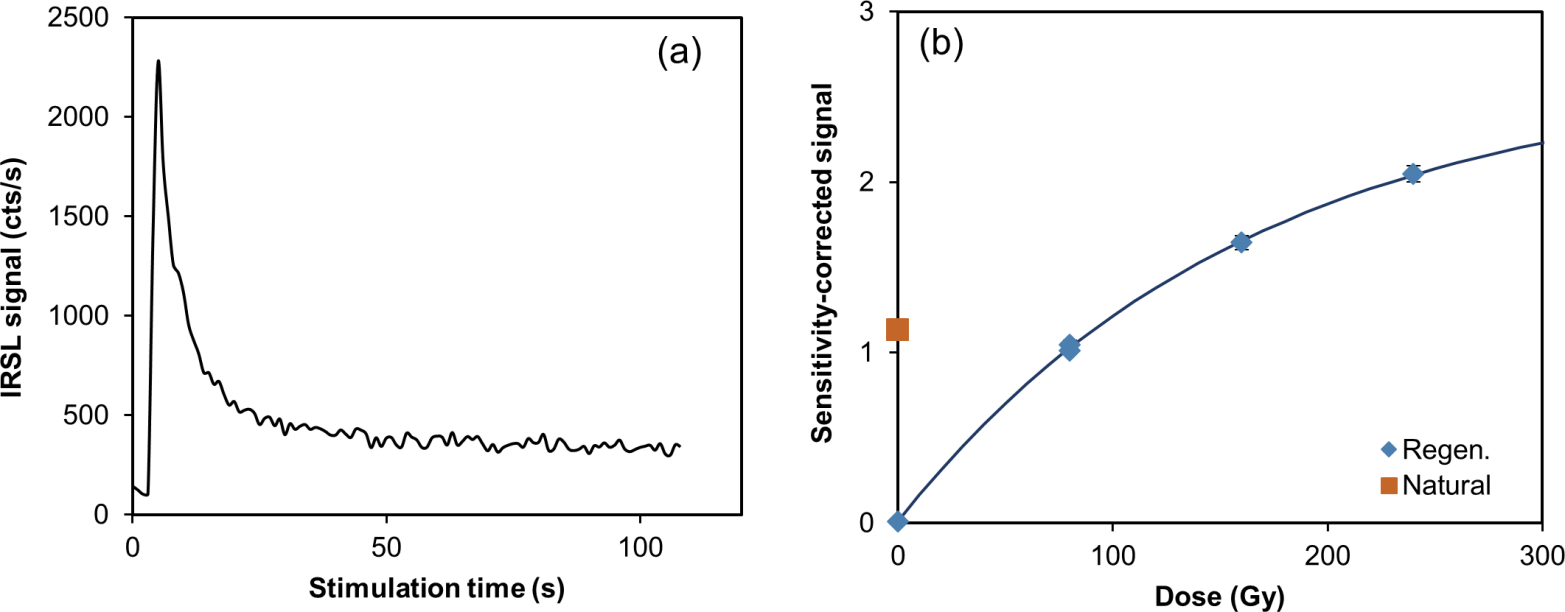
pIRIR decay curve and the corresponding dose-response curve for one aliquot from LB12-OSL3.

The pIRIR_290_ procedure was conducted on a total of seven samples collected from Leang Burung 2 in 2012 from Layer B (LB2-OSL13), Layer A (LB2-OSL12 & LB2-OSL11), Layer I (LB2-OSL10 & LB2-OSL6), Layer II (LB2-OSL4) and Layer V (LB2-OSL3). Examples of a pIRIR decay curve and the corresponding dose-response curve are shown in Fig 17 for one aliquot from LB12-OSL3. Similar results were obtained for the other samples. The dose-response curve for each aliquot was generated using a series of regenerative and test doses (the latter being used to monitor and correct for any sensitivity changes between measurement cycles), and the D_e_ is calculated by projecting its sensitivity-corrected natural signal on to this dose-response curve. Twelve aliquots were measured for each sample.

The performance of the pIRIR procedure was tested by measuring the residual dose and conducting a dose recovery test. To estimate the extent of any residual dose, we exposed four natural aliquots of each of the samples LB2-OSL3, OSL4, OSL6 and OSL10 to a solar simulator for ~4 hr and the remnant doses were measured using the pIRIR_290_ procedure. The residual doses obtained range from 6 to 9 Gy, and are consistent with each other at 2σ. These values were then subtracted from the D_e_ estimate to determine final ages for our samples. A weighted mean value of 7.3 ± 0.6 Gy was assumed for the other samples not measured in the residual dose test.

Similar to the residual dose measurement, four natural aliquots from each of LB2-OSL3 and OSL4 were bleached in a solar simulator for ~4 hr and then given a beta dose of 110 Gy. These aliquots were then measured using the same pIRIR_290_ procedure. A recovered ratio of 0.95 ± 0.02 and 0.96 ± 0.02 was obtained after correction for residual doses, indicating that the experimental conditions used here are able to recover the given dose.

**Fig 18:**
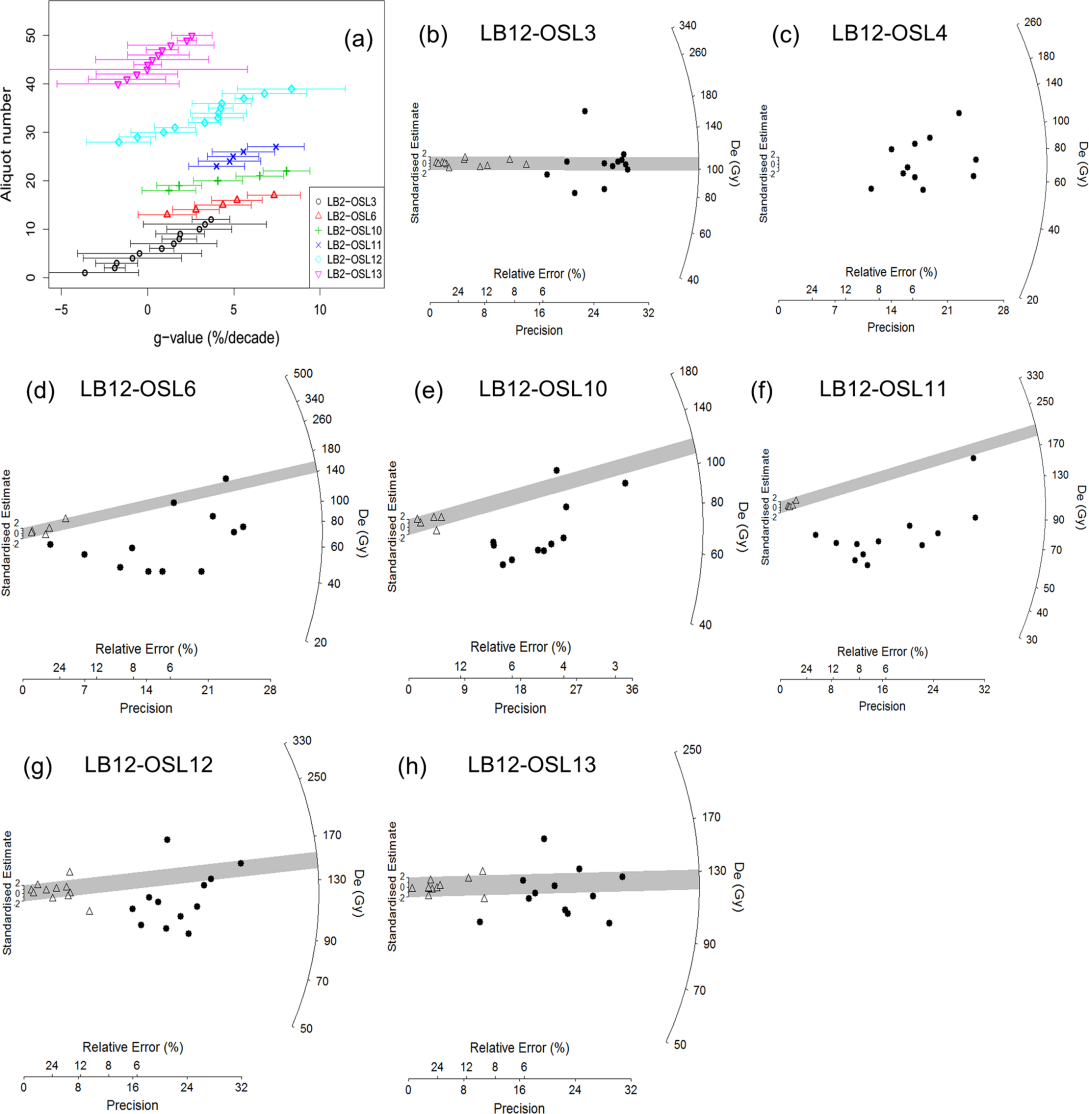
pIRIR data from Leang Burung 2. (**a**) Summary of the g-values obtained for individual aliquots from samples LB2-OSL3, LB2-OSL6, LB2-OSL10, LB2-OSL11, LB2-OSL12 and LB2-OSL13, respectively; (**b**-**h**) The fading-uncorrected (filled circles) and–corrected (triangles) D_e_ values for all the 2012 OSL samples shown in the radial plots.

As demonstrated previously [77], the feldspars from this region have a highly variable anomalous fading rate in the pIRIR signals, and a low temperature IR stimulation (at 50 or 100°C) used in a pIRIR procedure cannot completely remove the anomalous fading for the subsequent pIRIR signals. In order to test whether this is the case for our Leang Burung 2 samples, after D_e_ measurements, between five and 12 aliquots from each sample (except LB12-OSL4) were measured for an anomalous fading test. A single-aliquot measurement procedure similar to those described in [79], but based on the pIRIR procedure, was applied. The fading rates (g-values) were calculated for the pIRIR(50, 290) signal and normalised to the time of prompt measurement of the IRSL signal (t_c_ = 970 s). The g-values obtained are shown in Fig 18 for individual aliquots. It can be seen that, apart from LB12-OSL3, the g-values for which have a small range between -2 and 3%/decade, the other samples yielded a wide range of g-values (up to ~10%/decade) for different aliquots. This is similar to the observation obtained using the samples from Leang Bulu Bettue [77]. To account for this problem, the latter authors proposed a method to obtain reliable D_e_ estimates by extrapolation of the relationship between D_e_ and laboratory fading rate (g-value). Unfortunately, given the limited number of aliquots measured for g-value and D_e_ for our samples, it was not possible to apply that method to our samples. Instead, we used the fading correction method [80] for the aliquots with g-values measured, despite the large uncertainties associated with fading-corrected D_e_ values owing to the large uncertainty associated with the individual g-values.

The fading-uncorrected and fading-corrected D_e_ values for all the samples are shown in the radial plots in Fig 18B–18H, respectively. A central age model (CAM) [81] was applied to calculate the fading-uncorrected and–corrected D_e_ values, and these are summarised in Table 2. The CAM results for fading-corrected D_e_ values are shown as grey bands in the corresponding radial plots for each sample (Fig 18B–18H). It is shown that the fading-corrected CAM results are broadly consistent with the largest fading-uncorrected D_e_ values. This is similar to the observation of [77], who found that the non-fading or small-fading aliquots have the largest D_e_ values for their samples from Leang Bulu Bettue, implying that it is the variable fading rates among different aliquots that results in the large variation and different extents of underestimation in the D_e_ values. Hence, we have adopted the CAM results for the fading-corrected D_e_ values to estimate the final ages (Table 2).

The total dose rate for feldspar grains consists of four components: the external gamma, beta and cosmic-ray dose rates, and the internal beta dose rate. The external gamma dose rates were measured using an Exploranium GR-320 portable gamma-ray spectrometer, which is equipped with a 3-inch diameter NaI(Tl) crystal calibrated for U, Th and K concentrations using the CSIRO facility at North Ryde [82]. The external beta dose rate was measured by low-level beta counting using a Risø GM-25-5 multicounter system [83] and referenced to the Nussloch Loess (Nussi) standard. The external beta dose rate was corrected for the effect of grain size and hydrofluoric acid etching on beta-dose attenuation. These external components of the total dose rate were adjusted for assumed long-term water contents. These values are based on the measured field water contents, together with an assigned 1σ uncertainty of ± 5% (or ± 10% for the deepest samples) to capture the likely range of time-averaged values over the entire period of sample burial (Table 2). The dosimetry data for all samples are summarised in Table 2.

### U-series dating of faunal remains

We conducted laser ablation U-series dating of faunal remains from Leang Burung 2, following methods reported in detail in [60]. All U-series isotope analyses were measured using the laser ablation multi-collector inductively coupled mass spectrometer (MC-ICP-MS) system at The Australian National University’s (ANU) Research School of Earth Sciences. Sample details are provided in Table 3.

### U-series dating of straw stalactites

U-series dating of straw stalactites from Leang Burung 2 was carried out in the Radiogenic Isotope Facility of the University of Queensland, Australia, on a Nu Plasma MC-ICP-MS, followed procedures outlined in [55]. We dated a total of five straw stalactites collected at varying depths from Layers II, and a single specimen collected from Layer A (see Table 2 for sample details). The samples all consisted of small segments of broken straw stalactites, rather than whole or relatively complete examples of these fragile, ubiquitous speleothems. The straw stalactites were quite muddy and thus required careful pre-treatment for dating, including cutting, breaking into small chips, washing, hand-picking under a binocular microscope, and vigorous cleaning. Straw stalactites may grow over a long period of time [55]. Hence, for three of the samples, we dated both the tips and the bases of the straw stalactites separately. Where dated, the ‘tips’ of the broken stalactites were slightly younger than the ‘bases’, confirming their stratigraphic order and therefore the reliability of the U-series dates.
