# Correction: A reassessment of the early archaeological record at Leang Burung 2, a Late Pleistocene rock-shelter site on the Indonesian island of Sulawesi

**DOI:** 10.1371/journal.pone.0202357

**Published:** 2018-08-09

**Authors:** Adam Brumm, Budianto Hakim, Muhammad Ramli, Maxime Aubert, Gerrit D. van den Bergh, Bo Li, Basran Burhan, Andi Muhammad Saiful, Linda Siagian, Ratno Sardi, Andi Jusdi, Abdullah, Andi Pampang Mubarak, Mark W. Moore, Richard G. Roberts, Jian-xin Zhao, David McGahan, Brian G. Jones, Yinika Perston, Katherine Szabó, M. Irfan Mahmud, Kira Westaway, Jatmiko, E. Wahyu Saptomo, Sander van der Kaars, Rainer Grün, Rachel Wood, John Dodson, Michael J. Morwood

A portion of the figure legend for Fig 7 is incorrectly displayed in the second paragraph under the subheading “Evaluating Glover’s model of human occupation at the site 35–23 ka cal BP” in the Results section. Please see the complete, correct Fig 7 caption here.

**Fig 7:**
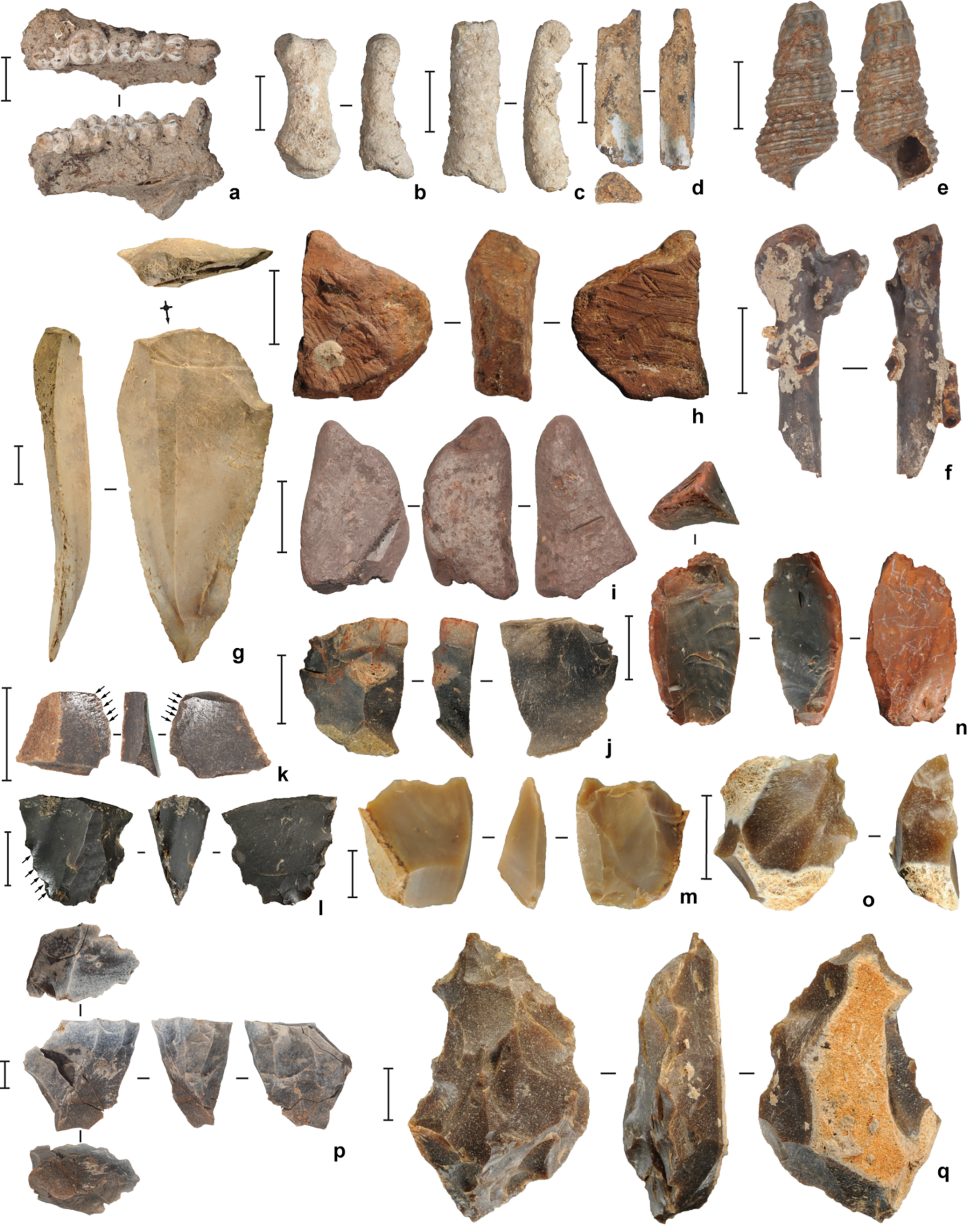
Faunal remains and artifacts excavated from the upper levels of square D11 at Leang Burung 2 in 2011. (**a**) *Macaca* sp. mandible, spit 31 (Layer II); (**b**-**c**) *Ailurops ursinus* (bear cuscus) phalanges; **b**, spit 42 (Layer II), **c**, spit 17 (Layer V); (**d**) *Macaca* sp. radius fragment (burnt), spit 46 (Layer II); (**e**) *Tylomelania* (= *Brotia*) *perfecta* shell (burnt), spit 19 (Layer II); (**f**) bird bone (burnt), spit 45 (Layer II); (**g**) chert macroblade point, spit 45 (Layer II); (**h**) utilised (faceted and scored) ochre piece, spit 45 (Layer II); (**i**) ochre ‘crayon’, spit 39 (Layer II); (**j**) chert flake with ochre residues, spit 30 (Layer II); (**k**-**l**) chert artifacts with silica gloss (highlighted by arrows); **k**, spit 18 (Layer V), **l**, spit 19 (Layer V); (**m**-**n**) chert bipolar cores; **m**, spit 19 (Layer V), **n**, spit 15 (Layer V); (**o**) retouched chert flake, spit 19 (Layer V); (**p**) single platform chert core, spit 45 (Layer II); (**q**) chert radial core, spit 13 (Layer V). Scale bars are 10 mm.
